# Identification and characterization of R75estA, a halophilic polyester-degrading enzyme from a marine bacterium

**DOI:** 10.3389/fmicb.2026.1870101

**Published:** 2026-07-10

**Authors:** Robert Ruginescu, Georgiana Necula-Petrareanu, Madalina Tudorache, Madalina Matei, Mihaela Muresan, Cristina Purcarea

**Affiliations:** 1 Research Institute of the University of Bucharest-ICUB, University of Bucharest, Bucharest, Romania; 2 Department of Microbiology, Institute of Biology Bucharest of the Romanian Academy, Bucharest, Romania; 3 Department of Inorganic and Organic Chemistry, Biochemistry and Catalysis, Faculty of Chemistry, University of Bucharest, Bucharest, Romania; 4 Department of Biology-Ecology, National Institute for Research and Development on Marine Geology and Geoecology-GeoEcoMar, Bucharest, Romania

**Keywords:** bioprospecting, Black Sea, cutinase, PET hydrolysis, polyesterase, *Stutzerimonas*, type III PETase

## Abstract

Polyester-based plastics, particularly polyethylene terephthalate (PET), represent a major fraction of global plastic waste, and their enzymatic recycling has attracted increasing attention as a sustainable alternative to conventional thermomechanical and chemical methods. However, the repertoire of characterized polyester-degrading enzymes, and particularly of the recently proposed type III PETases, remains limited. Here, we report the identification and biochemical characterization of R75estA, a polyesterase from *Stutzerimonas* sp. R75, isolated from the Black Sea during bioprospecting for polyester-degrading bacteria. Phylogenetic analysis placed R75estA as the first characterized member of a clade of *Stutzerimonas* and *Pseudomonas* homologs, while AlphaFold-based structural analysis predicted a canonical *α*/*β*-hydrolase fold with sequence and active-site features shared with type III PETases such as PmC, HaloPETase1, and dsPETase05. Biochemical characterization of the purified recombinant enzyme showed a halophilic, alkaliphilic, and moderately thermoactive hydrolase, with optimal activity at 3 M NaCl, pH 8.0, and 50 °C. R75estA hydrolyzed *p*-nitrophenyl esters of varying chain lengths, olive oil, polycaprolactone, bis(2-hydroxyethyl) terephthalate (BHET), and PET microparticles. To our knowledge, R75estA is the first polyesterase characterized from the genus *Stutzerimonas* and from the Black Sea, expanding the known sequence, structural, and ecological diversity of type III PETases.

## 1 Introduction

Synthetic polyesters are an important class of plastics used in a wide range of modern life applications, most notably in the textile and packaging sectors. They are characterized by ester bonds linking the repeating units of their macromolecular chains and include aromatic representatives such as polyethylene terephthalate (PET), aliphatic representatives such as polylactic acid (PLA) and polycaprolactone (PCL), and aliphatic–aromatic copolyesters such as poly(butylene adipate-co-terephthalate) (PBAT) (Urbanek et al., 2020). Unlike many types of plastics, which contain carbon–carbon backbones largely recalcitrant to biological degradation, ester bonds in polyesters are susceptible to enzymatic cleavage. Enzymatic recycling technologies for polyesters have therefore attracted growing attention as a more efficient and sustainable alternative to conventional thermomechanical and chemical recycling methods (Tournier et al., 2023). Enzymatic depolymerization operates under relatively mild conditions, eliminates the need for hazardous chemical catalysts, and can process contaminated, colored, and mixed polyester waste streams that are otherwise difficult to recycle. By recovering monomers to resynthesize virgin-quality polymers, this approach offers a promising pathway toward a circular economy for polyesters (Wei et al., 2025; Zimmermann, 2025).

Enzymes capable of cleaving ester bonds in polyesters, collectively referred to as polyesterases, are hydrolytic enzymes predominantly of bacterial and fungal origin, with some representatives also identified in archaea (Perez-Garcia et al., 2023; Tournier et al., 2023). The first characterized members of this group were cutinases (EC 3.1.1.74), named after their ability to hydrolyze cutin, the insoluble polyester that forms the protective cuticle of higher plants (Kawai et al., 2019). Beyond cutinases, polyester-hydrolyzing activity has since been identified across several subclasses of carboxylic ester hydrolases, including carboxylesterases (EC 3.1.1.1), lipases (EC 3.1.1.3), PETases (EC 3.1.1.101), and among some proteases (EC 3.4) (Tournier et al., 2023). Most of these enzymes belong to the polyesterase–lipase–cutinase family (Chatonnet et al., 2023) and share several structural features, including a central *β*-sheet flanked by *α*-helices, a Ser-His-Asp catalytic triad, and a solvent-accessible active site that typically lacks the hydrophobic lid characteristic of true lipases (Tournier et al., 2023). This open active-site topology allows polyesterases to hydrolyze a broad range of substrates, including short-chain esters, insoluble triglycerides, and polymers, without requiring interfacial activation (Urbanek et al., 2020).

Among polyesterases, PET hydrolases have been further subdivided into types I and II based on structural features such as the number and position of disulfide bonds, the amino acid composition of the active-site subsites, and the presence or absence of an extended loop surrounding the catalytic cleft (Joo et al., 2018). More recently, a third type has been proposed to include enzymes with significant structural divergence from the canonical types (Turak et al., 2025). Functionally, the three types display distinct biochemical properties and industrial applicability. Type I enzymes are typically thermostable, with melting temperatures (T_m_) above 70 °C, making them the most efficient PET hydrolases because PET is most effectively depolymerized near its glass transition temperature (Tournier et al., 2023). The leaf-compost cutinase LCC (Sulaiman et al., 2012), a type I enzyme further engineered for improved performance and thermostability, is currently used as the catalyst in the only PET recycling technology deployed on an industrial scale (Tournier et al., 2020). Type II enzymes, by contrast, are typically mesophilic (T_m_ < 55 °C) and are regarded as PET surface-modifying enzymes with promising applications for the hydrolytic treatment of textile fibers (Kawai et al., 2024). The most studied type II enzyme is IsPETase from *Piscinibacter sakaiensis* (formerly *Ideonella sakaiensis*), which has been engineered extensively to enhance activity and thermal stability, yielding variants with promising capabilities for recycling applications (Kawai et al., 2024). Type III enzymes, in turn, comprise a small group of moderately thermoactive enzymes (optimal temperature of ~50 °C) distinguished by their tolerance to high salt concentrations (3–5 M NaCl), a feature that may confer advantages for PET recycling under high-salinity industrial conditions (Turak et al., 2025).

The functional features of polyesterases are shaped by the physicochemical properties of the environments in which their microbial hosts live. Most polyesterases characterized to date have been obtained from terrestrial sources, such as soil, plant compost, and hot springs, whereas only a small fraction have been obtained from marine environments (Ruginescu and Purcarea, 2024). Marine polyesterases, however, frequently retain activity across wide ranges of temperature, pH, and salinity (Chen et al., 2024; Ruginescu and Purcarea, 2024; Turak et al., 2025), features that may prove advantageous under industrial conditions. Therefore, bioprospecting of underexplored marine habitats represents a promising strategy to expand the repertoire of polyesterases, which could contribute to the development of more efficient biocatalysts for biotechnological applications, particularly polyester recycling. In this context, the Black Sea represents a particularly interesting target: a semi-enclosed basin affected by substantial riverine inputs of plastic waste (Strokal et al., 2022) whose bacterial communities, despite showing documented esterolytic potential (Ruginescu et al., 2022), have not yet been explored for their polyesterase activity.

In this study, we screened a collection of bacterial strains isolated from the Black Sea for their ability to degrade aliphatic polyesters. *Stutzerimonas* sp. R75, one of the best-performing isolates, was selected for in-depth analysis of its polyester-degrading enzymes, leading to the identification and characterization of R75estA, a polyesterase that shares sequence and structural features with type III PET hydrolases. Biochemical characterization revealed a halophilic, alkaliphilic, and moderately thermoactive enzyme that hydrolyzes a broad range of substrates, including *p*-nitrophenyl esters, insoluble triglycerides, and synthetic polyesters. To our knowledge, R75estA represents the first polyesterase characterized from the genus *Stutzerimonas* and from the Black Sea, and these findings contribute to expanding the known sequence, structural, and ecological diversity of polyesterases.

## 2 Materials and methods

### 2.1 Isolation of marine bacterial strains

The bacterial strains screened in this study originated from two sources: strains previously isolated from the Romanian littoral zone of the Black Sea (Ruginescu et al., 2022), and strains isolated during the present study from surface seawater and sediment samples collected from nine sites in the western region of the Black Sea, at different depths and distances from the shoreline (Supplementary Figure S1; Supplementary Table S1). Collected samples were serially diluted in autoclaved seawater, previously filtered through 0.22 μM MF-Millipore membrane filters (Merck, Darmstadt, Germany), and 100 μL of each dilution was spread onto Enriched Seawater Agar (eSWA) (Ruginescu et al., 2022), MG50 (Karimi et al., 2019), Marine R2A diluted 1:10 (Suzuki et al., 1997), and WEM (Huang et al., 2021) media. Following a 7-day incubation period at 20 °C, colonies exhibiting distinct morphologies were purified by re-streaking onto Marine Agar (BD Difco, Franklin Lakes, NJ, USA) and subsequently stored at −80 °C in Marine Broth (Carl Roth GmbH, Karlsruhe, Germany) supplemented with 25% (v/v) glycerol.

### 2.2 Agar plate-based screening for polyesterase activity

Bacterial cultures were spot-inoculated in duplicate onto Marine Agar (diluted 1:2 in sterile seawater) supplemented with one of the following substrates (all from Sigma-Aldrich, St. Louis, MO, USA): 1.5% (v/v) tributyrin, 0.1% (w/v) polycaprolactone (PCL, M_n_ 10,000), or 1.5% (v/v) polycaprolactone diol (PCD, M_n_ 530). Substrate emulsions in sterile distilled water were prepared as previously described (Molitor et al., 2020) and mixed with the molten agar medium under continuous stirring. Inoculated plates were incubated at 20 °C for 9 days, followed by 14 days at 10 °C to enhance enzyme activity while slowing the bacterial growth rate. Hydrolytic activity was assessed as the ratio of the hydrolysis halo diameter to the bacterial spot diameter (in millimeters).

### 2.3 Genomic DNA extraction and 16S rRNA-based identification

Selected bacterial strains were cultivated in Marine Broth at 25 °C for 48–72 h. Cells from 1 mL of culture were harvested by centrifugation at 6,000 × *g* for 10 min, resuspended in 200 μL enzymatic lysis buffer (20 mM Tris–HCl, 2 mM sodium EDTA, 1.2% Triton X-100, 20 mg/mL lysozyme; pH 8.0), and incubated at 37 °C for 2 h. Genomic DNA was extracted from the cell lysate using a Quick-DNA Miniprep Plus Kit (Zymo Research, Irvine, CA, USA) according to the recommended workflow for bacterial samples. PCR amplification of the 16S rRNA gene was carried out in a 50 μL final reaction volume containing: 1x DreamTaq Green Master Mix (Thermo Fisher Scientific, Waltham, MA, USA), 0.2 μM primer 27F (Miller et al., 2013), 0.2 μM primer 1518R (Cui et al., 2009), 50 ng DNA template, and nuclease-free water. PCR conditions were as follows: 3 min denaturation at 95 °C, 30 cycles of 30 s denaturation at 95 °C, 30 s annealing at 55 °C, 90 s extension at 72 °C, and a final extension step of 5 min at 72 °C. Amplicons were purified using a DNA Clean & Concentrator-5 Kit (Zymo Research, Irvine, CA, USA) and sequenced by Macrogen Europe (Amsterdam, The Netherlands) using the amplification primers. The low-quality bases from the ends of the raw sequences were trimmed using CodonCode Aligner (v9.0.2), and the resulting sequences were compared with those in the GenBank refseq_rna database (NCBI) using BLASTn (Altschul et al., 1990).

### 2.4 Genome sequencing, assembly, and annotation

Whole-genome sequencing of *Stutzerimonas* sp. R75 was performed by Macrogen Inc. (Seoul, Republic of Korea) using the TruSeq DNA PCR-Free kit (350 bp insert size) for library preparation, followed by sequencing on the Illumina NovaSeq X platform with 150 bp paired-end reads. Raw sequencing reads were quality-checked using FastQC v0.12.1 (Babraham Bioinformatics, 2025), and adapter sequences along with low-quality bases were removed with Skewer v0.2.2 (Jiang et al., 2014). High-quality trimmed reads were assembled *de novo* using Unicycler v0.5.1 (Wick et al., 2017), with contigs shorter than 400 bp excluded. The quality of the final assembly was evaluated using QUAST v5.2.0 (Gurevich et al., 2013), while genome completeness and contamination were assessed with CheckM v1.0.18 (Parks et al., 2015). Genome annotation was performed using Prokka v1.14.6 (Seemann, 2014).

### 2.5 Genome mining for polyesterase-encoding genes

Amino acid sequences of 112 polyesterases with demonstrated activity against synthetic polyesters, primarily polyethylene terephthalate (Supplementary Table S2), were retrieved from the Plastics-Active Enzymes Database (PAZy) (Buchholz et al., 2022) and used to generate a custom reference database using the makeblastdb command-line application (Camacho et al., 2009). Potential polyesterases in the *Stutzerimonas* sp. R75 genome were identified by performing a BLASTp search of the Prokka-predicted proteins against the constructed database, using an e-value threshold of 1e-30.

### 2.6 Cloning of putative polyesterase-encoding genes

The genes encoding the enzymes R75estA (GenBank accession: MGR6587169.1) and R75estB (MGR6587921.1) were synthesized with codon optimization for expression in *Escherichia coli* and inserted individually into the pET22b(+) expression vector (Novagen, Madison, WI, USA) by Gene Universal Inc. (Newark, DE, USA). Cloning was performed using the *NdeI* (5′ end) and *XhoI* (3′ end) restriction sites, ensuring an in-frame fusion with a C-terminal hexa-histidine tag. Signal peptides were predicted using SignalP v6.0 (Teufel et al., 2022) and excluded from the synthesized DNA sequences. The recombinant plasmids were subsequently transformed into chemically competent *E. coli* BL21(DE3) (New England Biolabs, Ipswich, MA, USA) for protein production.

### 2.7 Recombinant protein production and purification

A freshly transformed *E. coli* BL21(DE3) colony was grown at 37 °C for 4 h with shaking (180 rpm) in LB broth (Miller) (Scharlab, Barcelona, Spain) supplemented with 0.5% (w/v) glucose and 100 μg/mL ampicillin. The culture was then diluted 1:200 in ZYM-5052 auto-induction medium (Studier, 2014) and incubated with shaking (180 rpm) for 23 h at 21 °C. Cells were harvested by centrifugation (6,000 × *g*, 10 min, 4 °C), resuspended in lysis buffer (50 mM sodium phosphate, 300 mM NaCl, 20 mM imidazole; pH 8.0), and disrupted by sonication on ice. The soluble fraction obtained after centrifugation (15,000 × *g*, 30 min, 4 °C) was mixed with pre-equilibrated Ni-NTA resin (Thermo Fisher Scientific, Waltham, MA, USA) for 1 h at 4 °C and subsequently loaded onto a gravity-flow column. The resin was washed with two bed volumes of lysis buffer supplemented with 60 mM imidazole, and His-tagged proteins were eluted with the same buffer containing stepwise increasing concentrations of imidazole (100, 150, 200, and 300 mM). Eluted fractions were analyzed by SDS-PAGE to assess protein purity and by activity assays on PCL agar plates. Fractions containing the target protein were pooled and desalted using Zeba Spin desalting columns (Thermo Fisher Scientific, Waltham, MA, USA). The protein was concentrated by ultrafiltration on Amicon Ultra 10 K filter devices (Merck, Darmstadt, Germany) and exchanged into storage buffer (20 mM HEPES, 150 mM NaCl, and 20% glycerol, pH 8.0). The concentration of the purified protein was determined spectrophotometrically at 280 nm based on the theoretical molar extinction coefficient (*ε* = 47,120 M-1 cm-1) calculated from the amino acid sequence using the ExPASy ProtParam server (Gasteiger et al., 2005). The final protein preparations were stored at −20 °C.

### 2.8 Enzyme phylogenetic and structural analyses

The amino acid sequence of R75estA was queried against the NCBI non-redundant (nr) protein database using BLASTp (Altschul et al., 1990), and sequences with greater than 90% similarity were downloaded for phylogenetic analysis. Multiple sequence alignment was performed in MEGA11 (Tamura et al., 2021) with MUSCLE, and a maximum-likelihood phylogenetic tree was subsequently constructed using the LG + F model with gamma-distributed rates among sites and 500 bootstrap replicates. Amino acid sequence alignments were visualized using ESPript 3.0 (Robert and Gouet, 2014). The three-dimensional (3D) structure of R75estA was predicted with AlphaFold 3 (Abramson et al., 2024) and visualized in UCSF ChimeraX v1.9 (Meng et al., 2023). Molecular docking of bis(2-hydroxyethyl) terephthalate (BHET) to R75estA was carried out using AutoDock Vina v1.2.0 (Eberhardt et al., 2021) implemented in SwissDock (Bugnon et al., 2024). The ligand structure was obtained from the ZINC database (ID: ZINC02040111) (Irwin et al., 2020). The search space was set as a cubic box (25 Å per side) centered on the catalytic triad, with an exhaustiveness parameter of 64. Docking poses were visualized and analyzed using UCSF ChimeraX v1.9.

### 2.9 Enzyme biochemical characterization

#### 2.9.1 Standard esterase activity assay

Esterase activity of purified R75estA enzyme was determined using *p*-nitrophenyl butyrate (*p*NPB) (Sigma-Aldrich, St. Louis, MO, USA) as substrate. Unless otherwise indicated, reactions were carried out in a total volume of 200 μL containing 0.8 mM *p*NPB (from a 100 mM stock in DMSO) in 50 mM sodium phosphate buffer with 150 mM NaCl, pH 8.0. Reactions were initiated by adding 2 μL of enzyme to a final concentration of 20 nM after a 5-min pre-equilibration at 30 °C. Hydrolysis of *p*NPB at 30 °C was monitored by measuring the release of *p*-nitrophenol (*p*NP) at 410 nm every 30 s for 5 min using a FLUOstar Omega microplate spectrophotometer (BMG Labtech, Ortenberg, Germany). Reaction mixtures lacking enzyme served as blanks to correct for non-enzymatic hydrolysis. The amount of *p*NP released was determined from a standard curve generated with known concentrations of *p*NP. One enzyme unit (U) was defined as the amount of enzyme required to release 1 μmol of *p*NP per minute under the assay conditions. Specific activity was expressed as units per milligram of protein (U/mg). All experiments included three technical replicates, and data are presented as mean ± standard deviation.

#### 2.9.2 Substrate specificity

Substrate specificity was determined using *p*-nitrophenyl esters of different chain lengths (all from Sigma-Aldrich, St. Louis, MO, USA): *p*NP-butyrate (*p*NPB; C_4_), pNP-octanoate (*p*NPO; C_8_), *p*NP-laurate (*p*NPL; C_12_), and *p*NP-palmitate (*p*NPP; C_16_). Substrate stocks were prepared in acetonitrile at 100 mM concentration for *p*NPB and *p*NPO, and 40 mM for *p*NPL and *p*NPP. Each reaction contained 0.8 mM substrate in 50 mM HEPES (pH 8.0), 0.25% (w/v) Triton X-100, 2% (v/v) acetonitrile, and 20 nM enzyme. The release of *p*NP at 30 °C was monitored every 20 s for 6 min for *p*NPB and *p*NPO, every 30 s for 20 min for *p*NPL, and every 60 s for 40 min for *p*NPP. All other conditions were consistent with those described in Section 2.9.1.

#### 2.9.3 Kinetic parameters

Kinetic constants (K_m_ and k_cat_) were determined by measuring reaction rates across a range of substrate concentrations: 0.2–2.8 mM for *p*NPB and *p*NPO, and 0.05–2.4 mM for *p*NPL. Each reaction mixture contained the substrate (prepared from stocks in acetonitrile) in 50 mM HEPES buffer (pH 8.0), 0.5% (w/v) Triton X-100, and 8% (v/v) acetonitrile. The final enzyme concentration was 10 nM for reactions with *p*NPB and 20 nM for those involving *p*NPO and *p*NPL. The release of *p*NP at 30 °C was monitored every 20 s for 7 min for *p*NPB and *p*NPO, and every 60 s for 60 min for *p*NPL. All other experimental parameters were identical to those of the standard assay described in Section 2.9.1. Initial velocities, obtained from triplicate measurements, were fitted to the Michaelis–Menten equation using non-linear regression analysis in GraphPad Prism (version 9.3.0) to calculate the kinetic parameters.

#### 2.9.4 Temperature profile and stability

To determine the effect of temperature on enzyme activity, standard assays were performed at temperatures ranging from 5 °C to 70 °C. For temperatures outside the microplate reader range (below 25 °C or above 50 °C), reactions were incubated on thermoblocks or in incubators for 5 min, and absorbance was measured immediately afterward. Thermal stability was evaluated by incubating the enzyme (2 μM in storage buffer) at 30, 40, 50, and 60 °C for 30 min to 168 h, followed by determination of residual activity under standard assay conditions.

#### 2.9.5 pH profile and stability

To assess the effect of pH on enzyme activity, esterase activity was measured across a pH range of 3.0 to 10.0 using a modified version of the standard assay. The reaction mixtures contained 0.8 mM *p*NPB and one of the following buffers (50 mM): sodium citrate (pH 3.0–6.0), sodium phosphate (pH 7.0–8.0), Tris–HCl (pH 8.5), or CHES (pH 9.0–10.0). The release of *p*NP was monitored for 5 min at the isosbestic point of 348 nm to ensure that absorbance measurements were independent of the *p*NP ionization state at different pH values. For pH stability analysis, the enzyme (2 μM) was incubated in the same buffers at 20 °C for up to 168 h. At selected time intervals, aliquots were withdrawn, and residual esterase activity was determined using the standard assay (pH 8.0).

#### 2.9.6 Salinity profile and stability

To evaluate the effect of salinity on enzyme activity, assays were performed in 100 mM HEPES (pH 8.0) supplemented with NaCl at concentrations of 0, 0.5, 1, 2, 3, 4, and 5 M. Enzyme stability at varying salinities was assessed through two experiments. For short-term stability, enzyme aliquots (2 μM) were incubated for 1 h at 25 °C in 100 mM HEPES containing 0, 1, 2, 3, or 4 M NaCl. For long-term stability, aliquots were incubated for 24, 96, and 168 h at 40 °C under identical buffer and salt conditions. At each time point, residual activity was quantified under standard assay conditions.

#### 2.9.7 Effect of chemical reagents on enzyme activity and stability

The influence of various chemicals, including metal ions (Ca^2+^, Mg^2+^, Mn^2+^, Ni^2+^, Cu^2+^, Zn^2+^, Fe^2+^, Fe^3+^, Al^3+^, and Hg^2+^), reducing agents (β-Mercaptoethanol (β-ME) and 1,4-dithiothreitol (DTT)), detergents (sodium dodecyl sulfate (SDS) Triton X-100, and Tween 80), and inhibitors (ethylenediaminetetraacetic acid (EDTA) and phenylmethylsulfonyl fluoride (PMSF)) was evaluated through two experiments. First, to assess the immediate effect on enzyme activity, the chemicals were added directly to the reaction mixture under standard assay conditions (Section 2.9.1) at final concentrations of 1 mM for ions, reducing agents, and inhibitors, or 0.1% (w/v) for detergents. Second, to assess stability, the enzyme (2 μM) was pre-incubated with these reagents at 25 °C for 1 h at higher concentrations: 10 mM for ions, 5 mM for reducing agents and inhibitors, and 1% w/v for detergents. Following incubation, residual activity was determined under standard assay conditions and compared to an untreated control.

### 2.10 Enzymatic hydrolysis of BHET and PET-mp

Commercial bis(2-hydroxyethyl) terephthalate (BHET) and laboratory-pretreated polyethylene terephthalate microparticles (PET-mp) were evaluated as substrates for the R75estA enzyme. PET-mp were obtained by solvent dissolution and reprecipitation using a modified protocol based on Vogel et al. (2021). Specifically, 1 g of a post-consumer PET bottle was dissolved in 20 mL of phenol at 90 °C for 2 h, and the solution was reprecipitated by the controlled addition of 100 mL of distilled water (1 mL/min) under stirring (1,500 rpm). The resulting microparticles were recovered by centrifugation (2,500 × *g*, 30 min), subjected to multiple washes with deionized water to remove residual phenol, and dried at 40 °C for 48 h.

Hydrolysis assays were performed by incubating 50 μL of R75estA enzyme (221.5 μg/mL) with 2 mg of substrate (either BHET or PET-mp) in 250 μL of sodium phosphate buffer (50 mM, pH 8.0) supplemented with 3 M NaCl. Reactions were maintained at 30 °C under continuous stirring for 7 days. For each substrate, control reactions lacking enzyme were incubated in parallel under identical conditions to account for non-enzymatic substrate hydrolysis, and the corresponding background was subtracted from the enzymatic reactions. After incubation, the reaction products were analyzed by HPLC-DAD using a Poroshell 120 EC-C18 column (4.6 × 50 mm, 2.7 μm), following the approach previously reported by Ion et al. (2021). The enzymatic efficiency, expressed as turnover frequency (TOF) for BHET and PET-mp degradation, was calculated according to the following formula:


$$\mathrm{TOF}\;(h^{-1})=\frac{\text{mass of the consumed substrate}\;(\mathrm{\mu g})}{\text{mass of the biocatalyst}\;(\mathrm{\mu g})\;x\;\text{reaction time}\;(h)}$$

Product selectivity was calculated as the percentage of each identified product relative to the total amount of released products.

## 3 Results

### 3.1 Bioprospecting the Black Sea for polyesterase-producing bacterial strains

Surface seawater and sediment samples collected from nine offshore stations in the Western Black Sea yielded microbial colonies with diverse morphologies when inoculated onto marine-optimized culture media. Based on distinct colony characteristics, 212 isolates from seawater (Supplementary Table S3) and 160 from sediments (Supplementary Table S4) were selected and screened for extracellular esterase activity, along with 35 previously obtained strains from the Black Sea littoral zone (Ruginescu et al., 2022). Screening on tributyrin-containing agar plates demonstrated that 374 (92%) of the 407 tested isolates produced extracellular esterases capable of hydrolyzing this short-chain triglyceride. The level of enzyme activity (LEA), estimated as the ratio of the hydrolysis halo diameter to the colony diameter, varied substantially among the isolates. While most exhibited weak (LEA < 1.2) and moderate (LEA < 1.8) activity, 115 isolates (28%) showed strong tributyrin hydrolysis, with LEA values generally exceeding 1.8 (Supplementary Table S5). These high-activity isolates were subsequently screened for polyesterase activity using polycaprolactone (PCL) and polycaprolactone diol (PCD) as model substrates, since the ability to hydrolyze these synthetic aliphatic polyesters may indicate potential for degrading more recalcitrant semiaromatic polymers such as polyethylene terephthalate (PET) (Molitor et al., 2020). This screening led to the identification of 55 isolates with PCL-degrading activity, 39 isolates capable of degrading PCD, and 30 isolates with hydrolytic activity on both substrates. Similar to the results obtained with tributyrin, enzyme activity on PCL and PCD varied among isolates, with LEA values ranging from less than 1.2 (barely detectable halos) up to 4.3 (large hydrolysis zones), suggesting differences in polyesterase secretion levels and/or catalytic efficiency (Supplementary Table S5). Notably, three isolates demonstrated complete hydrolysis of PCL, as indicated by the formation of clear halos around their colonies, whereas the remaining isolates produced semitransparent halos, suggesting incomplete polymer breakdown.

Taxonomic identification based on 16S rRNA gene sequencing was conducted on 17 of the best-performing isolates (LEA > 1.5) that exhibited PCL and/or PCD-degrading activity. These isolates were affiliated with diverse genera within the classes Gammaproteobacteria, Bacilli, and Actinomycetes (Table 1). Among them, isolates R75, GS26, and EN12, identified as the most potent polyesterase producers based on agar plate screening, showed high sequence identity to species within the genera *Stutzerimonas*, *Nocardiopsis*, and *Isoptericola*, respectively.

**Table 1:** Identification and polyesterase activity of a subset of Black Sea bacterial isolates.

Strain	16S rRNA-based Identification			Hydrolysis of:		
	Genus and species (% identity)	Class	Accession no.	Tributyrin	PCL	PCD
W31	*Pseudoalteromonas haloplanktis* (99.6)	Gammaproteobacteria	PV765531	+	+ (p)	+
G2	*Pseudoalteromonas prydzensis* (99.1)	Gammaproteobacteria	PV765532	+	+ (p)	+
W3	*Pseudoalteromonas prydzensis* (99.1)	Gammaproteobacteria	PV765533	+	+ (p)	−
W63	*Pseudoalteromonas prydzensis* (99.1)	Gammaproteobacteria	PV765534	+	+ (p)	−
R64	*Pseudoalteromonas prydzensis* (99.2)	Gammaproteobacteria	PV765535	+	+ (p)	−
W80	*Rheinheimera aestuarii* (99.6)	Gammaproteobacteria	PV765536	+	+ (p)	+
R88	*Rheinheimera aestuarii* (99.8)	Gammaproteobacteria	PV765537	+	+ (p)	+
R75	*Stutzerimonas zhaodongensis* (99.8)	Gammaproteobacteria	PV765538	+	+	+
R83	*Shewanella vesiculosa* (99.9)	Gammaproteobacteria	PV765539	+	+ (p)	+
WS42	*Pseudomonas leptonychotis* (99.7)	Gammaproteobacteria	PV765540	+	+ (p)	+
RS21	*Alteromonas portus* (99.3)	Gammaproteobacteria	PV765541	+	+ (p)	+
GS15	*Peribacillus frigoritolerans* (99.8)	Bacilli	PV765542	+	+ (p)	+
WS55	*Peribacillus frigoritolerans* (100)	Bacilli	PV765543	+	+ (p)	+
EN45	*Peribacillus simplex* (99.9)	Bacilli	OL672374	+	+ (p)	+
WS32	*Bacillus* sp. (99.7)	Bacilli	PV765544	+	+ (p)	−
GS26	*Nocardiopsis exhalans* (99.3)	Actinomycetes	PV765545	+	+	−
EN12	*Isoptericola halotolerans* (99.6)	Actinomycetes	OL672343	+	+	+


PCL, polycaprolactone; PCD, polycaprolactone diol; p, partial hydrolysis. Unless otherwise stated, (+) indicates complete substrate hydrolysis, and (−) denotes no detectable enzyme activity.

### 3.2 Identification of putative polyesterases in the *Stutzerimonas* sp. R75 genome

**Figure 1:**
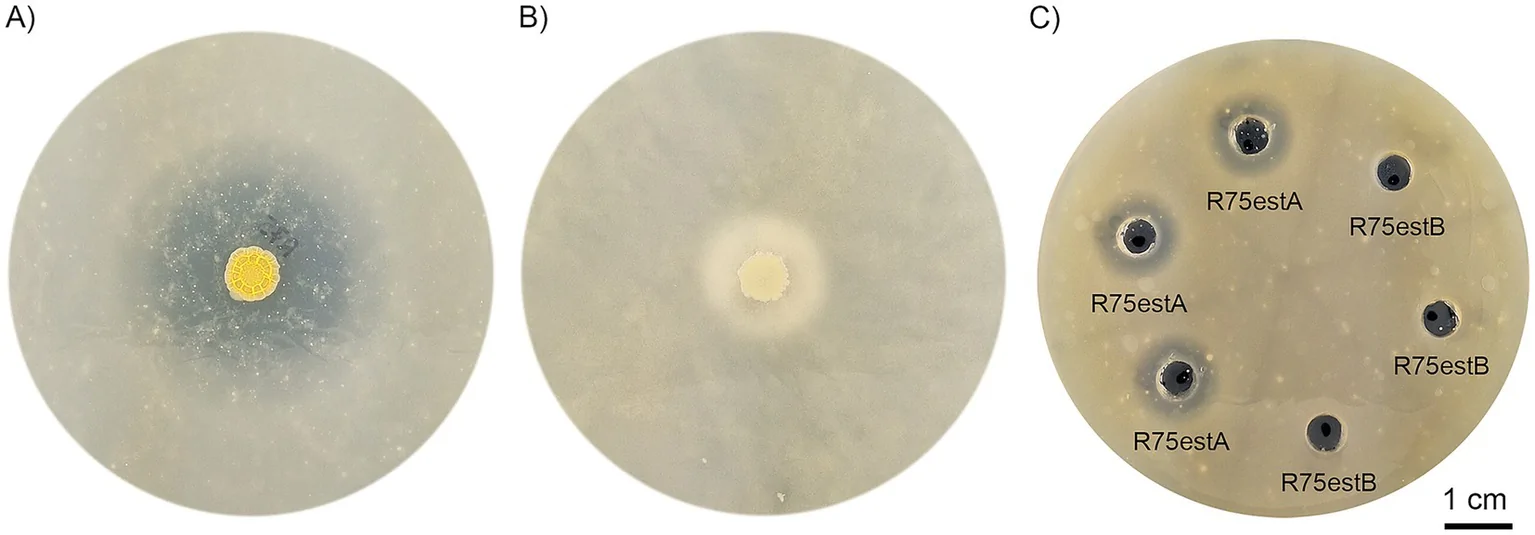
Hydrolysis of polycaprolactone substrates by *Stutzerimonas* sp. R75 and recombinant *E. coli* crude extracts. **(A,B)** Strain R75 grown on agar plates containing **(A)** polycaprolactone (PCL) and **(B)** polycaprolactone diol (PCD), showing hydrolysis activity evidenced by a clear halo on PCL and a precipitation halo on PCD after 7 days of incubation at 25 °C; **(C)** Hydrolysis of PCL by crude enzyme extracts from *E. coli* expressing R75estA (positive hydrolysis) and R75estB (no hydrolysis), as evidenced by the clear zone around the wells containing R75estA after overnight incubation at 40 °C.

The marine bacterial strain *Stutzerimonas* sp. R75, which exhibited strong aliphatic polyester-degrading activity (Figures 1A,B), was selected for *in silico* identification of polyesterase-encoding genes. To this end, genomic DNA was sequenced using the Illumina NovaSeq X platform, and the resulting high-quality data (Q30 92.8%) were assembled into 39 contigs, achieving 100% genome completeness and minimal contamination (0.14%) as determined by CheckM. The assembled genome spanned 4.81 Mb, had a high GC content of 60.3%, and contained 4,422 coding sequences (CDS), consistent with other *Stutzerimonas* genomes available in the NCBI database (Supplementary Table S6).

Mining of the *Stutzerimonas* sp. R75 genome, through alignment of Prokka-predicted proteins with 112 experimentally validated synthetic polyester-degrading enzymes (Supplementary Table S2), led to the identification of three putative polyesterases: R75estA (GenBank accession: MGR6587169.1), R75estB (MGR6587921.1), and R75estC (MGR6588980.1). These enzymes showed significant sequence identity (Table 2) to carboxylic ester hydrolases from *Pseudomonadaceae* representatives, including PmC (Ronkvist et al., 2009b), dsPETase05 (Chen et al., 2024), HaloPETase1 (Turak et al., 2025), PpEst (Haernvall et al., 2017; Wallace et al., 2017), and EstB (Edwards et al., 2022). In addition, a BLASTp search of the three R75 enzymes against the NCBI protein database demonstrated full or very high sequence identity (>90%) with several uncharacterized proteins from *Stutzerimonas* and *Pseudomonas* genomes, predominantly of marine origin (Supplementary Table S7).

**Table 2:** Homology of *Stutzerimonas* sp. R75 proteins to known synthetic polyester-degrading enzymes based on BLASTp alignment.

R75 protein	Homologous protein	Microbial source of homologous protein	Identity (%)	Alignment length (aa)	E-value	Bit score
R75estA	PmC	*Pseudomonas mendocina* ATCC 53552	80.4	281	1.95e-165	453
	dsPETase05	Pseudomonadota bacterium	61.5	281	8.51e-127	356
	HaloPETase1	*Halopseudomonas* lineage	65.2	256	3.68e-126	353
R75estB	PpEst (tesA)	*Pseudomonas pseudoalcaligenes* DSM 50188	68.6	201	4.40e-101	283
R75estC	EstB	*Pseudomonas* sp. strain 9.2	70.9	217	8.53e-116	322

aa = amino acids.

To further discriminate which of the three enzymes is involved in PCL and potentially PET hydrolysis, their amino acid sequences were analyzed for the presence of signal peptides indicative of extracellular secretion. SignalP 6.0 analysis predicted high-confidence Sec/SPI signal peptides in R75estA and R75estB, with cleavage sites between residues 23–24 and 21–22, and associated probabilities of 93.7 and 97.7%, respectively (Supplementary Figure S2). In contrast, R75estC lacked a detectable signal peptide, suggesting cytoplasmic localization and a potentially complementary role in polyester degradation, possibly involving intracellular processing of degradation intermediates. Based on the predicted extracellular localization, R75estA and R75estB were selected for cloning and recombinant expression.

### 3.3 Identification of R75estA as an active polyesterase

To functionally validate the two putative extracellular polyesterases identified *in silico*, R75estA and R75estB were heterologously expressed in *E. coli* using auto-induction conditions. Since the predicted signal peptides were excluded from the cloning constructs, the recombinant enzymes were retained intracellularly. Crude enzyme extracts were obtained by cell lysis followed by centrifugation to collect the soluble protein fraction. These extracts were then screened for PCL-degrading activity on agar plates containing emulsified PCL. Clear degradation zones were observed for R75estA, indicating active polyesterase function, whereas R75estB showed no detectable activity under the same conditions (Figure 1C). As a result, only R75estA was chosen for purification and further biochemical characterization in the present study.

### 3.4 Phylogenetic analysis and structural prediction of R75estA

**Figure 2:**
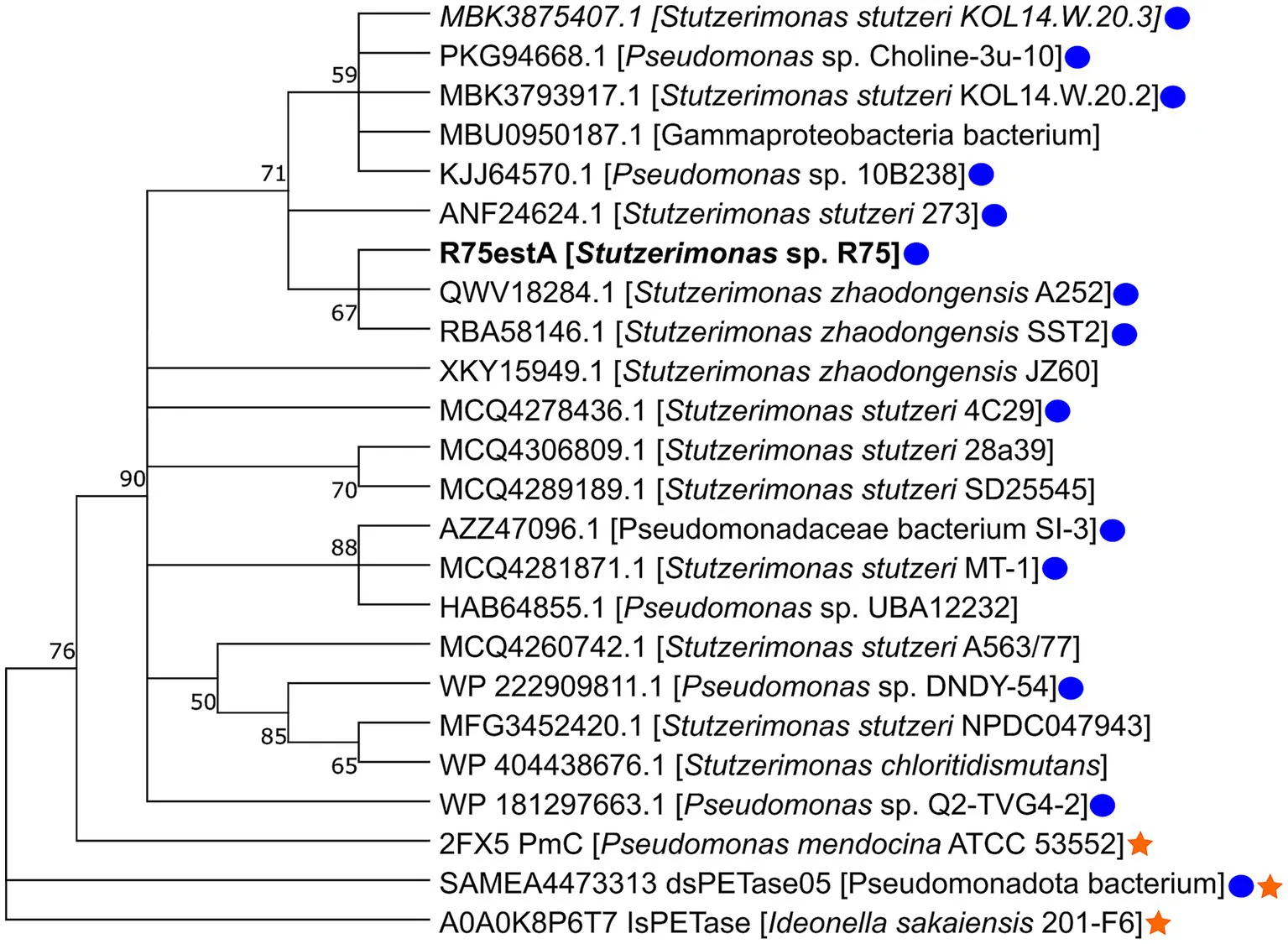
Phylogenetic tree of R75estA and homologous amino acid sequences identified in the NCBI database, rooted with IsPETase (NCBI accession: A0A0K8P6T7) as the outgroup. Phylogenetic relationships were inferred using the maximum-likelihood method, and the tree was condensed by collapsing branches with bootstrap values below 50% from 500 replicates. Blue circles indicate strains of marine origin, while orange stars denote enzymes with confirmed activity against synthetic polymers.

To identify close relatives of R75estA, its amino acid sequence was searched against the NCBI protein database using BLASTp. The analysis identified several highly similar sequences (>90% identity) from *Stutzerimonas* and *Pseudomonas* strains (Supplementary Table S7). In a maximum-likelihood phylogenetic analysis, these sequences clustered with R75estA into a distinct, well-supported clade (bootstrap value of 90%) (Figure 2). The closest functionally characterized homolog located outside this clade is PmC from *Pseudomonas mendocina* (80% sequence identity), a cutinase with reported activity on PET (Ronkvist et al., 2009b), polyvinyl acetate (Ronkvist et al., 2009a), and polybutylene succinate (Hu et al., 2024). Additionally, R75estA shares moderate identity (62–65%) with HaloPETase1 and dsPETase05, two almost identical enzymes active on PET (Chen et al., 2024; Turak et al., 2025). Notably, none of the sequences within the immediate R75estA clade (>90% identity) have been structurally or functionally characterized.

**Figure 3:**
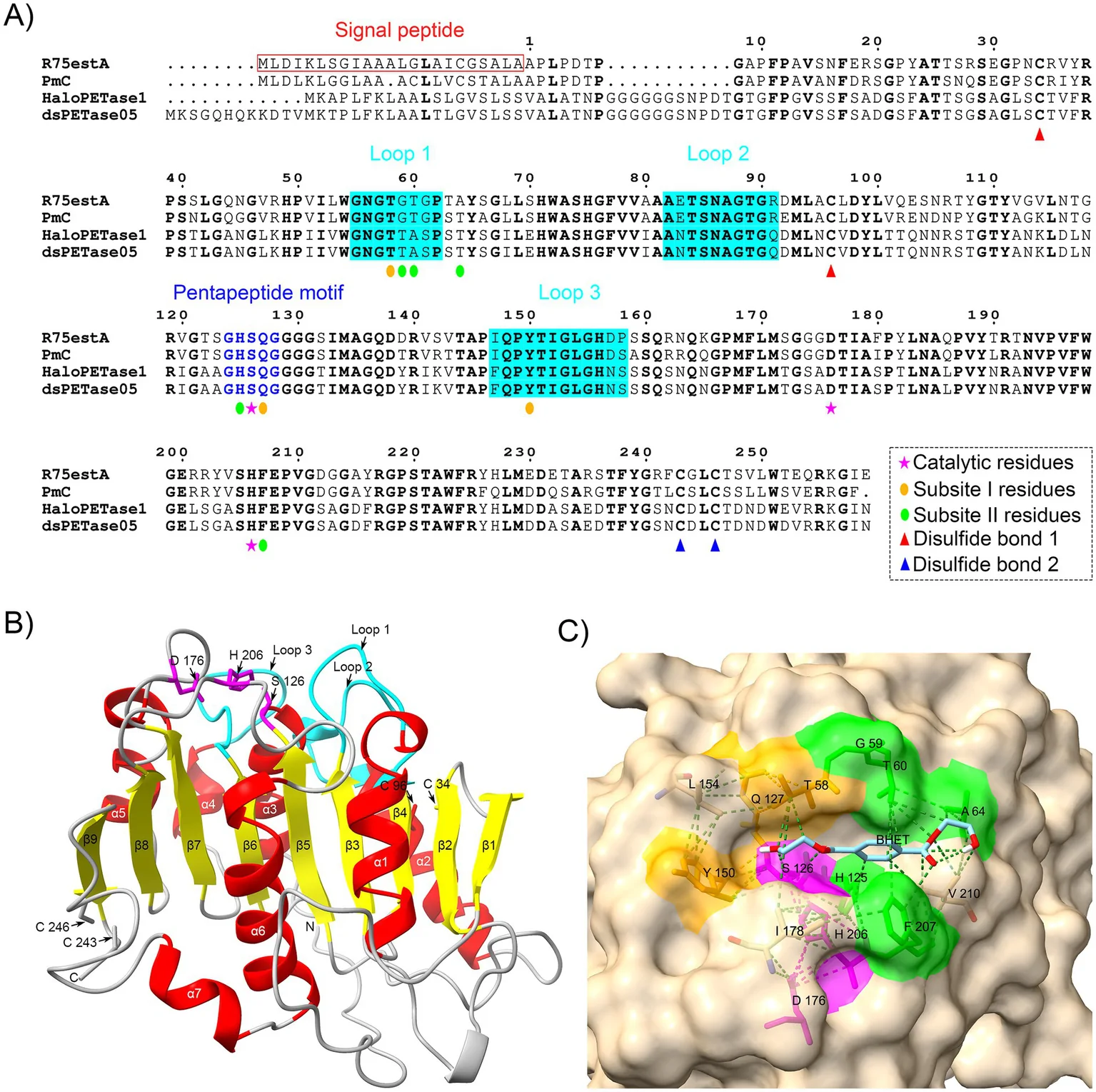
Structural analysis of R75estA. **(A)** Multiple sequence alignment with homologous proteins. Conserved residues are shown in bold. **(B)** Predicted 3D structure highlighting *β*-strands (yellow), *α*-helices (red), catalytic triad residues (magenta sticks), loops (grey/cyan), and disulfide bond residues (sticks). **(C)** Surface representation of the active site showing the predicted docking pose of BHET. Subsite I residues are colored orange, subsite II residues green, and the catalytic triad residues are shown in magenta.

Analysis of the multiple sequence alignment between R75estA and structurally characterized homologs (PmC, dsPETase05, and HaloPETase1) (Figure 3A) identified a Gly-X₁-Ser-X₂-Gly pentapeptide motif at the active site, as well as a putative Ser-His-Asp catalytic triad, two features generally conserved among α/β-hydrolases active on plastics (Buchholz et al., 2022). In R75estA, the pentapeptide motif contains His125 and Gln127 at the X₁ and X₂ positions, respectively, as in the other aligned homologs recently proposed to form the type III PETase group (Turak et al., 2025), distinct from the canonical type I, IIa, and IIb enzymes (Joo et al., 2018). In addition, four cysteine residues were identified in R75estA, suggesting the potential formation of two disulfide bridges: one located in the N-terminal region (Cys34-Cys96) and another near the C-terminus (Cys243-Cys246). The positions of these predicted bonds are conserved among the aligned type III PET hydrolases but differ from those observed in type II enzymes, in which one disulfide bridge is located near the active site (Joo et al., 2018).

The predicted 3D structure of R75estA, generated from its amino acid sequence using AlphaFold (mean Cα pLDDT = 98.6; pTM = 0.97), exhibits an α/β-hydrolase fold similar to that of most characterized plastic-degrading enzymes (Bauer et al., 2020; Buchholz et al., 2022), which are generally classified within the polyesterase–lipase–cutinase family (Chatonnet et al., 2023). It displays a central β-sheet composed of nine mostly parallel β-strands flanked on both sides by seven α-helices, with the β-strands and α-helices connected by relatively short loops (Figure 3B), a structural arrangement closely resembling that of the crystallographically resolved PmC enzyme (Boston et al., 1997). The predicted catalytic triad Ser126-His206-Asp176 is located on the protein surface and is solvent-accessible, a common feature among plastic-degrading enzymes of the polyesterase–lipase–cutinase family (Bauer et al., 2020).

Analysis of both the amino acid sequence and the predicted 3D structure indicated that, in addition to the conserved pentapeptide motif, catalytic triad, and disulfide bridges, R75estA shares several substrate-binding site features with type III PETases, notably the composition of key residues forming subsites I and II and the presence of equivalent loop regions that shape the active site cleft (Figure 3A). Subsite I is predicted to be formed by Thr58, Gln127, and Tyr150, residues that are conserved and essential for enzymatic activity in PmC (Bott et al., 2003), HaloPETase1 (Turak et al., 2025), and dsPETase05 (Zhang et al., 2025). Subsite II is predicted to comprise the highly conserved residues His125 and Phe207, which are present in all type III PETases, along with less conserved residues Gly59, Thr60, and Ala64. Docking simulations with BHET suggested that four of these residues (all except Gly59) directly contribute to substrate binding (Figure 3C).

The amino acid composition and predicted 3D structure of the loops defining the active site architecture of R75estA (Figures 3A,B) closely resemble those of homologous type III PETases. Three loop regions (loops 1–3) surround the active-site cleft, and their lengths and residue compositions differ from those of the corresponding loops in type I and type II enzymes, as recently described for HaloPETase1 (Turak et al., 2025) and dsPETase05 (Zhang et al., 2025). Most notably, loop 3 in R75estA contains a five-residue insertion (Gly153-Leu154-Gly155-His156-Asp157) that is absent from type I and type II PETases but conserved among all aligned type III enzymes.

### 3.5 Biochemical characterization of R75estA

Recombinant R75estA, fused with a C-terminal hexa-histidine tag, was purified to homogeneity by immobilized metal affinity chromatography, yielding approximately 0.5 mg of enzyme per liter of culture. SDS-PAGE analysis of the purified fraction showed a single protein band at approximately 30 kDa (Supplementary Figure S3), which is in good agreement with the theoretical molecular mass of the recombinant protein (i.e., 28,992.17 Da).

**Figure 4:**
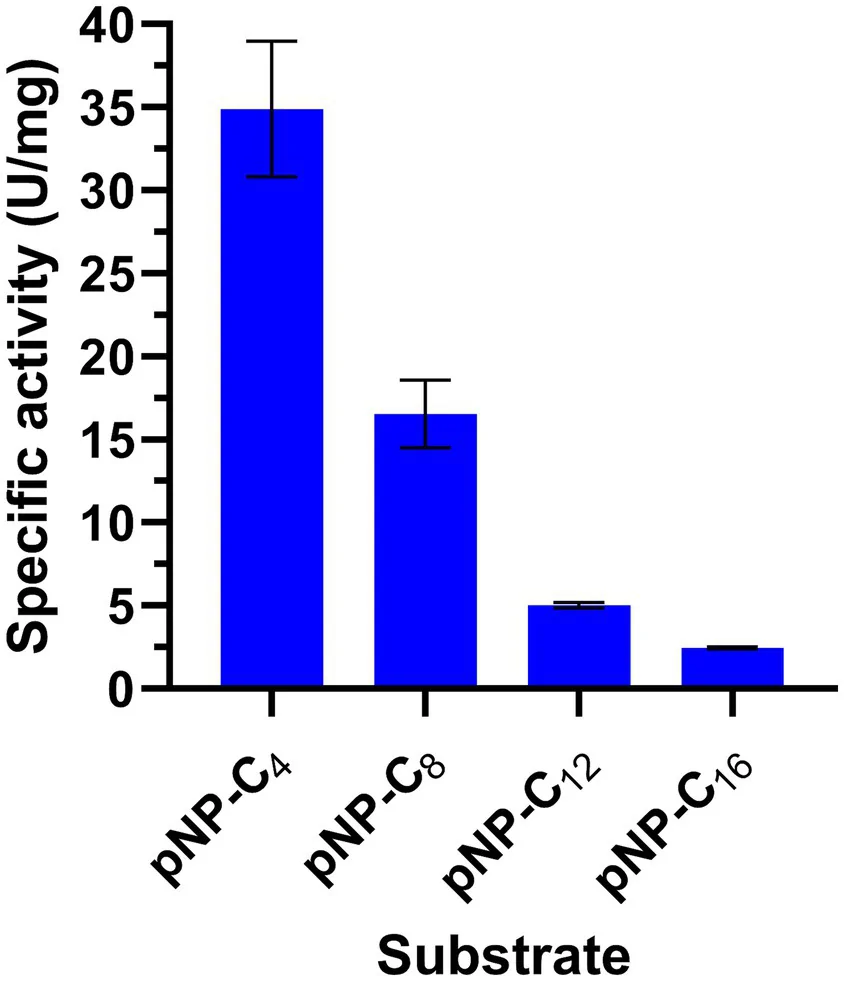
Substrate specificity of R75estA. The enzymatic activity was determined at 30 °C in 50 mM HEPES (pH 8.0) containing 0.8 mM substrate, 0.25% (w/v) Triton X-100, 2% (v/v) acetonitrile, and 20 nM enzyme. Data represent the mean ± standard deviation (SD) of three replicates.

The substrate specificity of R75estA was determined using *p*-nitrophenyl esters with acyl chain lengths ranging from C_4_ to C_16_. The enzyme exhibited maximal activity toward *p*NP-C_4_ (34.9 ± 4.0 U/mg). Activity decreased significantly as the acyl chain length increased, with 43% of the maximal activity for *p*NP-C_8_, 13% for *p*NP-C_12_, and 6% for *p*NP-C_16_ (Figure 4). Despite this preference for short-chain esters, R75estA also hydrolyzed olive oil in agar plate assays, confirming its lipolytic capacity (Supplementary Figure S4).

To determine the catalytic efficiency of R75estA, its kinetic parameters were further investigated using *p*NP-C_4_, -C_8_, and -C_12_ (Table 3). The enzyme exhibited the highest turnover rate (k_cat_) toward *p*NP-C_4_ (31.22 ± 2.51 s^−1^), which was approximately three- and five-fold higher than the rates observed for *p*NP-C_8_ and *p*NP-C_12_, respectively. The lowest Michaelis constant (K_m_), indicating the highest substrate affinity, was detected for *p*NP-C_8_. Consequently, the catalytic efficiency (k_cat_/K_m_) was highest for *p*NP-C_4_, followed closely by *p*NP-C_8_. A substantial decline in efficiency was observed for *p*NP-C_12_, primarily determined by the reduction in the turnover rate as the acyl chain length increased. Given its superior catalytic efficiency, *p*-nitrophenyl butyrate (*p*NP-C_4_) was selected as the model substrate for all subsequent biochemical characterization experiments.

**Table 3:** Michaelis–Menten kinetic parameters of R75estA toward *p*-nitrophenyl esters^a^.

Substrate	K_m_ (mM)	k_cat_ (s^−1^)	k_cat_/K_m_ (mM^−1^ s^−1^)
*p*NP-C_4_	1.06 ± 0.21	31.22 ± 2.51	29.29 ± 3.73
*p*NP-C_8_	0.43 ± 0.07	10.57 ± 0.49	24.35 ± 3.34
*p*NP-C_12_	0.92 ± 0.18	5.602 ± 0.48	6.04 ± 0.73

a All data are presented as the mean ± standard error (SE) of three replicates.

**Figure 5:**
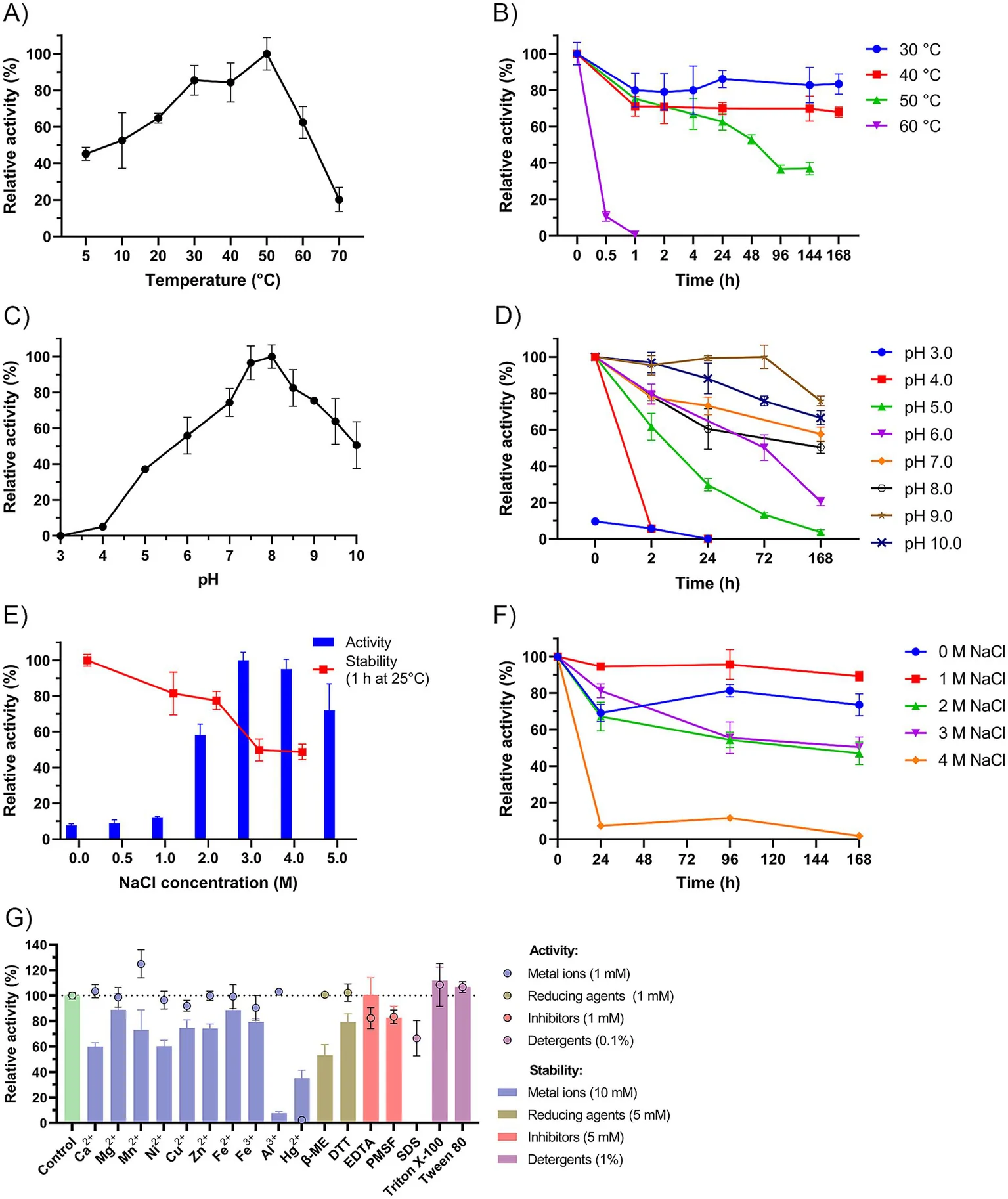
Functional characterization of R75estA. The effect of temperature on **(A)** enzyme activity and **(B)** stability. The influence of pH on **(C)** enzyme activity and **(D)** stability. The effect of NaCl concentration on **(E)** enzyme activity and short-term stability (1 h incubation at 25 °C) and **(F)** long-term stability (168 h incubation at 40 °C). **(G)** The effect of metal ions, reducing agents, inhibitors, and detergents on enzyme activity and stability. All data points represent the mean ± standard deviation (SD) of three replicates. Relative activity is expressed as a percentage of the maximum activity observed (for optimums) or the initial activity (for stability), unless otherwise stated.

The effect of temperature on R75estA activity was determined in the range of 5–70 °C. The enzyme exhibited maximum hydrolytic activity at 50 °C (Figure 5A) yet retained substantial activity at low temperatures, showing approximately 45% relative activity at 5 °C. In contrast, activity declined sharply at temperatures above 50 °C, reaching approximately 20% at 70 °C, presumably due to thermal denaturation. The thermal stability was investigated over 168 h at temperatures between 30 and 60 °C. R75estA exhibited high stability at 30 °C and 40 °C, retaining approximately 83 and 68% residual activity, respectively, at the end of the incubation period (Figure 5B). At the optimal temperature (50 °C), the enzyme exhibited a half-life of approximately 48 h, while at 60 °C, activity dropped to 10% within 30 min and was undetectable after 1 h.

The influence of pH on enzyme activity was assessed over the pH range of 3.0–10.0. R75estA showed an optimum at pH 8.0 (Figure 5C) and remained highly active in the alkaline range, with over 75% of its maximal activity at pH 9.0 and approximately 50% at pH 10.0. In contrast, activity dropped sharply in acidic conditions, with negligible activity (5%) at pH 4.0 and complete inactivation at pH 3.0. Regarding pH stability, R75estA retained more than 50% residual activity across the range of pH 7.0–10.0 over 168 h (Figure 5D). The highest stability was observed at pH 9.0, with approximately 75% residual activity, followed by 66% at pH 10.0. On the other hand, the enzyme was highly sensitive to acidic environments; at pH 4.0, more than 90% of activity was lost within 2 h, while at pH 5.0, inactivation was nearly complete after 168 h.

The effect of salinity on the catalytic activity of R75estA was investigated using NaCl concentrations ranging from 0 to 5 M. Relative activity was low (8–12%) between 0 and 1 M NaCl, but increased markedly at concentrations exceeding 1 M, with a peak of 100% at 3 M NaCl (Figure 5E). This maximum represents an approximately 12-fold enhancement over the activity recorded in salt-free conditions. Although hydrolytic activity remained high at 4 M NaCl (95%), it declined to 72% at 5 M. To further determine the impact of salinity on functional stability, R75estA was assessed under both short- and long-term incubation conditions. Following short-term incubation (1 h at 25 °C), maximal activity was preserved in the absence of salt; consequently, this condition served as the 100% reference (Figure 5E). Stability declined progressively as NaCl concentration increased, with relative activity decreasing to approximately 50% after incubation in 3–4 M NaCl. Long-term stability assays (168 h at 40 °C) identified 1 M NaCl as the optimal condition, where 89% of the initial activity was retained (Figure 5F). The enzyme also demonstrated significant stability in the absence of salt, retaining 73% relative activity after 168 h. In contrast, the hypersaline environments that favored rapid catalysis proved more detrimental to long-term stability: while the enzyme retained approximately 50% activity at 2–3 M NaCl, incubation at 4 M NaCl resulted in nearly complete inactivation within 24 h.

The influence of various metal ions, inhibitors, and detergents on the immediate activity and short-term stability of R75estA was investigated (Figure 5G). When cations were introduced into the reaction mixture at a final concentration of 1 mM, most exerted a negligible effect on enzyme performance, maintaining activity levels comparable to the control. Notably, Mn^2+^ appeared to enhance the initial catalytic rate by approximately 25%. To further characterize this effect, R75estA activity was measured over a broad range of Mn^2+^ concentrations (0–100 mM; Supplementary Figure S5). The activation was concentration-dependent and biphasic, reaching a maximum of approximately 128% at 0.5 mM Mn^2+^ and declining to baseline (~95–98%) at concentrations of 2 mM and above. The saturable activation at sub-millimolar concentrations suggests specific binding at a defined site rather than a non-specific ionic effect. A comparable behavior has been reported for the cutinase Cut190, in which Mn^2+^ binds at peripheral, non-catalytic sites and modulates the enzyme’s conformation (Senga et al., 2019). Unlike Cut190, however, which requires divalent cations for activity, R75estA remains active in their absence, indicating that Mn^2+^ acts as a modulator of activity rather than as an obligatory cofactor. The activity decline at higher concentrations may reflect a second, lower-affinity interaction that counteracts this activation, in line with the reduced stability observed in the presence of 10 mM Mn^2+^, where residual activity declined to approximately 70% after 1 h incubation at 25 °C. A detailed structural characterization would be required to explain the mechanistic basis of this Mn^2+^ effect. A major discrepancy between activity and stability was observed for Al^3+^. While the immediate addition of 1 mM Al^3+^ did not inhibit catalysis, a 1 h incubation at 10 mM resulted in a near-total loss of function (residual activity <10%). In contrast, Hg^2+^ functioned as a strong immediate inhibitor, with activity falling below 3%, yet the enzyme retained approximately 35% of the untreated control activity after the incubation period. This suggests that Hg^2+^-induced inhibition may be partially reversible or significantly less disruptive to the overall protein fold than the destabilizing effects of Al^3+^. The other tested metal ions also reduced enzyme stability, with residual activities ranging from 60 to 88% in the following decreasing order of stability: Mg^2+^ > Fe^2+^ > Fe^3+^ > Cu^2+^ > Zn^2+^ > Ca^2+^ > Ni^2+^. The addition of 1 mM EDTA to the reaction mixture resulted in a reduction in catalytic activity by 18% compared with the control. However, the enzyme’s stability remained unaffected after 1 h incubation with a higher concentration of the chelator (5 mM). This discrepancy suggests that while R75estA is not strictly a metalloenzyme requiring metal ions for its structural integrity, certain divalent cations may be involved in optimizing its catalytic rate. Furthermore, the serine-specific inhibitor PMSF exerted an inhibitory effect on both immediate activity and stability, suggesting the presence of a functional serine residue within the enzyme’s catalytic triad.

The potential role of the predicted disulfide bonds was further assessed using the reducing agents *β*-mercaptoethanol (β-ME) and 1,4-dithiothreitol (DTT). Although these agents did not interfere with the immediate catalytic activity, a significant time-dependent destabilization was observed. Following 1 h incubation, residual activity dropped to 53% with β-ME and 79% with DTT. Given that R75estA contains four cysteine residues, these results are consistent with the predicted disulphide bonds being important for maintaining the active conformation in aqueous environments. A direct attempt to detect these bonds by SDS-PAGE under reducing and non-reducing conditions (Supplementary Figure S6) showed no difference in band mobility, likely because the predicted loops are too small to alter electrophoretic migration. Further analysis will be required to confirm the disulfide pairing.

The enzyme demonstrated high tolerance toward non-ionic detergents; both Triton X-100 and Tween 80 maintained or even slightly enhanced catalytic activity (107–108%). Moreover, the enzyme activity was not negatively affected after 1 h incubation with 1% (w/v) of these detergents. In contrast, the anionic detergent SDS acted as a strong denaturant. While the enzyme retained 66% of its activity when SDS was added directly to the reaction at 0.1% (w/v), a 1 h incubation at 1% resulted in a total loss of function.

### 3.6 Hydrolysis of BHET and PET-mp by R75estA

**Figure 6:**
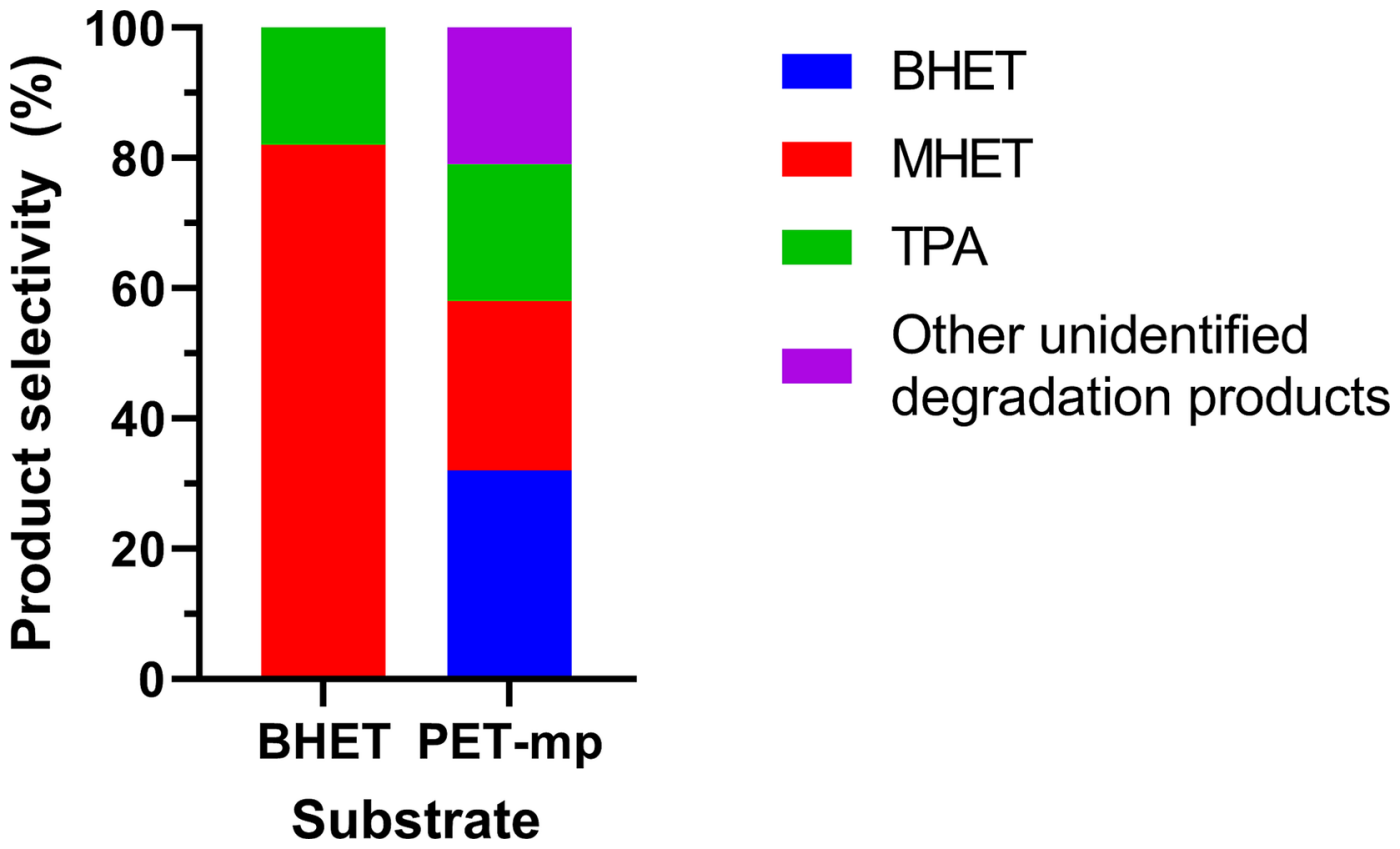
Product selectivity of R75estA-catalyzed hydrolysis of BHET and PET-mp. Reactions were carried out at 30 °C for 7 days in 50 mM sodium phosphate buffer (pH 8.0) containing 3 M NaCl. Product distribution is expressed as the percentage of each identified product relative to the total released products.

The ability of R75estA to hydrolyze bis(2-hydroxyethyl) terephthalate (BHET) and polyethylene terephthalate microparticles (PET-mp) was evaluated over 7 days at 30 °C under high-salinity conditions identified as optimal for enzyme activity (3 M NaCl). Detection and quantification of the degradation products by HPLC-DAD confirmed that R75estA hydrolyzed both substrates, with comparable turnover frequencies (TOF) of 3 × 10^−2^ h^−1^ for BHET and 2 × 10^−2^ h^−1^ for PET-mp. Controls without enzyme yielded no products for PET-mp and only trace background for BHET, which was subtracted from the reported values. Regarding product selectivity, MHET was the dominant product (82%) of BHET hydrolysis, followed by TPA (18%). In contrast, PET-mp degradation yielded a more heterogeneous product distribution, with BHET (32%), MHET (26%), and TPA (21%) as the main products (Figure 6).

## 4 Discussion

### 4.1 The Black Sea as a reservoir of polyesterase-producing microorganisms

Marine environments are increasingly recognized as promising reservoirs of microorganisms with polyester-degrading capabilities (Danso et al., 2018; Chen et al., 2024; Omura et al., 2024). Enzymes derived from these microorganisms frequently display niche-related catalytic features such as halotolerance, cold adaptation, or thermostability that distinguish them from their terrestrial counterparts and may prove advantageous for biotechnological applications (Ruginescu and Purcarea, 2024). Nevertheless, because enzyme discovery efforts have predominantly favored land-based sources such as compost, soil, and hot springs, marine-derived polyesterases still represent a small fraction of all characterized plastic-degrading enzymes (Ruginescu and Purcarea, 2024). Among the world’s marine environments, the Black Sea stands out as a particularly compelling yet underexplored target for bioprospecting polyesterase-producing microorganisms. Its semienclosed geography, combined with substantial riverine inputs of microplastics (Strokal et al., 2022), promotes the accumulation of plastic debris and may exert selective pressure on microbial communities, potentially favoring microorganisms with esterolytic capabilities. A previous enzyme screening of bacterial strains from the Romanian littoral zone demonstrated widespread extracellular esterase and carbohydrase activities among the cultured isolates but did not assess their capacity to degrade synthetic polyesters (Ruginescu et al., 2022). The present study extends that work by screening both the previously obtained littoral strains and a new collection of bacterial isolates from offshore Black Sea seawater and sediment samples for the ability to degrade aliphatic polyesters, thereby contributing to the growing repertoire of marine-derived polyesterase producers.

This screening identified three isolates with complete aliphatic polyester-degrading activity: *Stutzerimonas* sp. R75, *Nocardiopsis* sp. GS26, and *Isoptericola* sp. EN12. While our broader research aim is to characterize the enzymatic machinery of all three isolates, this work prioritizes *Stutzerimonas* sp. R75. Its classification within the *Pseudomonadaceae* aligns with the diverse capabilities for synthetic polymer degradation frequently reported in this family, particularly within the related genera *Pseudomonas* (Ronkvist et al., 2009b; Zhou et al., 2024), *Halopseudomonas* (Bollinger et al., 2020; Turak et al., 2025), and *Rhizobacter* (Sagong et al., 2021; Avilan et al., 2023). Notably, this study represents the first characterization of a polyesterase from the genus *Stutzerimonas*.

### 4.2 The polyesterase system of *Stutzerimonas* sp. R75

The identification of three putative polyesterases (R75estA, R75estB, and R75estC) within the *Stutzerimonas* sp. R75 genome suggests a multi-enzyme system adapted for ester-bond hydrolysis in marine environments. Our *in silico* analysis distinguished these enzymes by predicted cellular localization. Specifically, R75estA and R75estB are predicted to be secreted extracellularly via the Sec/SPI pathway, where they may initiate the depolymerization of high-molecular-weight polymers. In contrast, the absence of a signal peptide in R75estC points toward a cytoplasmic role, which may involve the hydrolysis of shorter oligomeric intermediates transported into the cell. Consistent with this interpretation, the homologous EstB enzyme (71% sequence identity with R75estC) has been shown to hydrolyze the PET degradation intermediate bis(2-hydroxyethyl) terephthalate (BHET), while exhibiting limited activity toward PET microparticles (Edwards et al., 2022). R75estC likely serves a similar intracellular function by processing smaller degradation products rather than intact polymers.

To assess the predicted extracellular polyesterase activities, both R75estA and R75estB were heterologously expressed in *Escherichia coli*. Although R75estA and R75estB share significant sequence similarity with known enzymes that degrade synthetic polyesters, functional screening using polycaprolactone (PCL) identified only R75estA as an active hydrolase. The lack of PCL activity for R75estB does not necessarily exclude a role in polyester catabolism, since the agar plate assay used here is qualitative and tests a single substrate under one set of conditions. Supporting this caution, the close relative PpEst (68% sequence identity) has been reported to degrade poly(1,4-butylene adipate-co-terephthalate) (PBAT) and specific ionic phthalic polyesters (Haernvall et al., 2017; Wallace et al., 2017), suggesting that R75estB may possess polyesterase activity against substrates and under conditions not tested here. These observations suggest that *Stutzerimonas* sp. R75 is likely to possess a substrate range that extends beyond the aliphatic polyesters tested in this study, and broader functional characterization of R75estB is an important direction for future work. However, the pronounced activity of R75estA toward PCL justified its selection as the primary candidate for the detailed biochemical characterization presented in this work.

### 4.3 Phylogenetic classification and structural features of R75estA

Phylogenetic analysis placed R75estA within a clade of closely related sequences from *Stutzerimonas* and *Pseudomonas* strains, none of which have been functionally or structurally characterized to date. This finding establishes R75estA as the first enzyme from this lineage to be studied in detail. Its closest characterized homolog, the cutinase PmC from *Pseudomonas mendocina* (80% sequence identity), has been shown to hydrolyze PET and other synthetic polyesters (Ronkvist et al., 2009a, 2009b; Hu et al., 2024). More distant homologs include HaloPETase1 and dsPETase05 (62–65% identity), two nearly identical enzymes independently discovered from marine metagenomes and reported to degrade PET under high-salinity conditions (Chen et al., 2024; Turak et al., 2025). The phylogenetic position of R75estA, together with the predicted structural features discussed below, supports its classification as a member of the recently proposed type III PETase group (Turak et al., 2025), which currently includes PmC, HaloPETase1, and dsPETase05.

The classification of PET hydrolases into types I, IIa, and IIb has been based on structural and sequence features near the active site, including the presence of extended loops, the number of disulfide bridges, and the amino acid composition at subsites I and II (Joo et al., 2018; Richter et al., 2023). R75estA does not conform to any of these established types. Instead, it shares a set of distinguishing features that have also been reported in HaloPETase1 (Turak et al., 2025), dsPETase05 (Zhang et al., 2025), and PmC (Boston et al., 1997). The first notable feature is the pentapeptide motif. In R75estA, this motif follows a Gly-His-Ser-Gln-Gly pattern, in which His at the second position is typical of type I PET hydrolases, whereas Gln at the fourth position replaces the Met residue highly conserved in type I and type II enzymes (Joo et al., 2018; Richter et al., 2023). Gln127 is predicted to participate in the formation of the oxyanion hole, together with Thr58, stabilizing the negatively charged tetrahedral intermediate generated during ester bond hydrolysis (Tournier et al., 2023). The functional importance of this residue is supported by mutagenesis data from dsPETase05, in which replacement of the equivalent Gln with the canonical Met (Q169M) resulted in a substantial loss of enzymatic activity (Zhang et al., 2025).

A second distinguishing feature of R75estA is the Tyr150 residue at subsite I, which replaces the Trp residue that forms part of a *π*-stacking clamp in type I and type II PETases. In those enzymes, two opposing aromatic residues (e.g., Tyr87 and Trp185 in IsPETase, or Phe63 and Trp156 in PHL7) flank the aromatic moiety of the PET substrate, stabilizing it through π-π interactions (Joo et al., 2018; Richter et al., 2023). In R75estA and other type III enzymes, this interaction is absent because the Trp-to-Tyr substitution is accompanied by a nonaromatic Thr (Thr58 in R75estA) at the opposing position. Mutagenesis experiments in both HaloPETase1 and dsPETase05 confirmed that the type III active site is incompatible with the classical π-stacking architecture: attempts to restore the clamp by introducing aromatic residues at the Thr58 position (T88W, T88F, T88Y in HaloPETase1; T100Y in dsPETase05) reduced activity by approximately 50% in HaloPETase1 (Turak et al., 2025) and 70% in dsPETase05 (Zhang et al., 2025). Moreover, substitutions of the residue equivalent to Tyr150 in dsPETase05 (Y192F, Y192W) reduced enzymatic activity by 65–80% relative to the wild-type enzyme (Zhang et al., 2025), confirming that this residue is essential for catalysis in type III PETases.

The positions of the two predicted disulfide bonds in R75estA further support its classification as type III. While type II PETases also harbor two disulfide bonds, one of these is located adjacent to the active site, where it plays an important role in maintaining active-site integrity (Fecker et al., 2018). In contrast, the predicted disulfide bridges in R75estA are positioned far from the active site, a pattern conserved among all type III enzymes (Turak et al., 2025; Zhang et al., 2025). This arrangement suggests that the disulfide bonds in type III PETases contribute primarily to global protein stability rather than to active site architecture. Mutagenesis data have shown that disruption of either disulfide bond in dsPETase05 by mutation (C138A or C285A) significantly diminished PET-hydrolyzing activity, with the C-terminal bond disruption being particularly detrimental (Zhang et al., 2025).

Another distinctive feature of type III PETases is the extended loop 3, which contains a five-residue insertion (Gly153-Leu154-Gly155-His156-Asp157 in R75estA). In R75estA, this loop is predicted to be situated near the active site, where, by analogy to HaloPETase1, it is expected to influence the shape of the substrate-binding cleft (Turak et al., 2025). Deletion mutagenesis on dsPETase05 demonstrated that sequential removal of residues from this loop led to a progressive decline in enzymatic activity, whereas alanine substitution mutagenesis identified His198 (equivalent to His156 in R75estA) as essential for catalysis (Zhang et al., 2025). Turak et al. (2025) further noted that Leu163 in HaloPETase1 (corresponding to Leu154 in R75estA), located on this extended loop, is positioned between the residues that would form a π-stacking clamp in other PETases, potentially blocking substrate insertion between the aromatic residues and thus contributing to a substrate-binding mode distinct from that described in type I and type II enzymes (Joo et al., 2018; Richter et al., 2023).

The analysis of the predicted structure also identified Phe207 in subsite II of R75estA as a promising target for future enzyme engineering. In dsPETase05, the substitution of the equivalent residue (Phe249) with Lys, Ile, or Leu yielded mutants with both enhanced enzymatic activity and increased thermostability, presumably by reducing steric hindrance and facilitating substrate access to the active site (Zhang et al., 2025). Similar beneficial substitutions at the equivalent position in PmC have also been reported (Bott et al., 2003). Given that Phe207 is conserved in R75estA, analogous mutations represent a rational starting point for improving its catalytic performance, particularly in combination with other engineering strategies such as disulfide bond introduction or machine learning–guided optimization, approaches that have proven effective for dsPETase05 (Zhang et al., 2025).

Although the structural features discussed above are supported by a high-confidence AlphaFold model, experimental structure determination, ideally in complex with a substrate or substrate analog, will be required to confirm the catalytic triad, the active-site architecture, and the substrate-binding interactions discussed here. Site-directed mutagenesis of the predicted catalytic residues, of key residues at the predicted subsites, and of the four cysteines forming the predicted disulfide bonds will provide complementary functional validation and will guide the rational engineering of R75estA.

### 4.4 Functional properties and halophilic adaptation of R75estA

The majority of enzymes capable of hydrolyzing plastics, including type I, II, and III PETases, belong to the polyesterase-lipase-cutinase family in the ESTHER database classification (Chatonnet et al., 2023). Members of this family typically exhibit the highest activity toward short-chain (C_4_) and medium-chain (C_6_-C_8_) *p*-nitrophenyl esters, although many also hydrolyze longer-chain substrates (C_12_-C_18_) as well as high-molecular-weight polyesters, both natural (e.g., cutin) and synthetic (e.g., PCL, PET) (Chen et al., 2013; Sulaiman et al., 2014). This broad substrate range distinguishes polyesterases from classical esterases, which are generally restricted to soluble short-chain esters, and from true lipases, which preferentially act on insoluble long-chain triacylglycerols (Fojan, 2000). The substrate specificity of R75estA aligns with this pattern: the enzyme exhibited maximal activity toward *p*NP-C_4_, with progressively declining activity as acyl chain length increased. This preference for short-chain substrates is reflected in the kinetic parameters, with the highest turnover rate (k_cat_) and catalytic efficiency (k_cat_/K_m_) detected for *p*NP-C_4_. Notably, the lowest Michaelis constant (K_m_) was observed for *p*NP-C_8_, indicating higher substrate affinity for this medium-chain ester; however, the reduced turnover rate at this chain length resulted in a catalytic efficiency comparable to that of *p*NP-C_4_. In addition, R75estA hydrolyzed olive oil, demonstrating lipolytic capacity, as well as synthetic aliphatic (PCL) and aromatic (PET) polyesters, supporting its classification as a polyesterase.

The catalytic features of R75estA are broadly in line with those reported for other marine-derived polyesterases (Ruginescu and Purcarea, 2024), which function optimally at moderate temperatures (i.e., 30–50 °C), at neutral to alkaline pH (i.e., 7.0–9.5), and in a few cases under high-salinity conditions (i.e., 1.5–5.3 M NaCl). A comparison among selected marine polyesterases is provided in Table 4. Notably, HaloPETase1 and dsPETase05, both type III PETases with significant sequence identity to R75estA, showed optimal activity at 3 and 5.3 M NaCl, respectively. Two additional marine polyesterases, PET6 and SM14est, also exhibited salt-enhanced activity, albeit at lower NaCl concentrations (0.9–1.5 M). Whether PmC, the closest characterized homolog of R75estA, shares a comparable salt preference remains unknown, as the effect of NaCl concentration on its activity has not been investigated.

**Table 4:** Optimal reaction conditions of marine-derived polyesterases.

Enzyme	Source	Temperature (°C)	pH	NaCl concentration (M)	Reference
R75estA	*Stutzerimonas* sp.	50	8.0	3	This study
PmC	*Pseudomonas mendocina*	50	9.5	ND (0)^a^	Ronkvist et al. (2009a, 2009b)
HaloPETase1	*Halopseudomonas* sp.	50	9.5–10.0	3	Turak et al. (2025)
dsPETase05	Pseudomonadota bacterium	55	ND (9.0)^a^	5.3	Chen et al. (2024)
PET6	*Vibrio gazogenes*	50	ND (8.5)^a^	1–1.5	Weigert et al. (2022)
SM14est	*Streptomyces* sp.	40–50	8.5–9.0	0.9	Carr et al. (2023); Carletti et al. (2025)
MtCut	*Marinactinospora thermotolerans*	45	8.0–8.5	ND (0.5)^a^	Liu et al. (2022)
PET27	*Aequorivita* sp.	40	7.0–8.0	ND (0)^a^	Zhang et al. (2022)
Rcut	*Rhodococcus* sp.	40	9.0	ND (0)^a^	Won et al. (2022)

a The optimal catalytic property has not been determined (ND); the values in parentheses indicate the conditions under which the enzymatic assays were carried out.

The halophilic character of R75estA may be explained by its relatively high content of acidic amino acids. Halophilic enzymes typically exhibit a high proportion of surface-exposed aspartate and glutamate residues, which counteract the destabilizing effects of high ionic strength by facilitating the retention of a hydration shell around the protein (Gunde-Cimerman et al., 2018). In line with this principle, Zhang et al. (2025) demonstrated that the C-terminal enrichment of acidic residues is essential for the halophilic properties of dsPETase05, in which 18 acidic residues were identified, 10 of which are located in the C-terminal region following the last catalytic triad residue. Domain-swapping experiments, in which the C-terminal segment of dsPETase05 was replaced with the corresponding region from IsPETase, resulted in enhanced activity under salt-free conditions but diminished halotolerance, while the reciprocal swap conferred salt tolerance on IsPETase chimeras. Sequence analysis of R75estA revealed a comparable total number of acidic residues (9 Asp and 10 Glu), of which 7 are located in the C-terminal region following His206 (Supplementary Figure S7). This value is between that of dsPETase05 and IsPETase (5 C-terminal acidic residues; NaCl optimum 0 M) and correlates with the intermediate NaCl optimum of R75estA (3 M). Although these observations suggest a model in which the proportion of C-terminal acidic residues modulates the degree of salt preference, the limited number of characterized halophilic PETases precludes definitive conclusions.

### 4.5 The potential of R75estA as a biocatalyst in PET recycling

The preliminary assays using BHET and PET-mp as substrates confirmed that R75estA is capable of hydrolyzing both compounds under high-salinity conditions. The detection of BHET, MHET, and TPA among the PET-mp degradation products is in line with the sequential hydrolysis pathway commonly reported for various PET-degrading enzymes (Ronkvist et al., 2009b; Sulaiman et al., 2012; Turak et al., 2025), in which BHET and MHET are generated as intermediates before their further conversion to TPA. However, the modest turnover frequencies observed (2–3 × 10^−2^ h^−1^) and the use of a single set of reaction conditions highlight the preliminary nature of these results. A dedicated study focused on optimizing substrate loading, enzyme concentration, reaction temperature, and salt concentration will be necessary to fully evaluate the catalytic potential of R75estA toward PET.

The current benchmark for enzymatic PET depolymerization is the engineered LCC variant ICCG, which achieves 90% conversion of post-consumer PET waste within approximately 10 h at 72 °C (Tournier et al., 2020). This performance is enabled by operating near the glass transition temperature of PET (approximately 70–80 °C), at which polymer chain mobility increases significantly, making the substrate far more accessible to enzymatic attack (Tournier et al., 2023). R75estA, with a temperature optimum of 50 °C and rapid thermal inactivation above 60 °C, operates well below this threshold, a limitation shared by most marine PETases described to date (Ruginescu and Purcarea, 2024). In line with this, the specific PET-hydrolysis activity of LCC and its engineered variants at 65 °C is approximately three orders of magnitude higher than that observed for R75estA on PET-mp at 30 °C (Tournier et al., 2020). By contrast, R75estA’s activity is of the same order of magnitude as that reported for unengineered IsPETase and FsC at 40 °C (Tournier et al., 2020), placing it within the activity range of PET hydrolases operating at moderate temperatures (30–50 °C). However, these direct comparisons should be interpreted with caution, given the differences in substrate form, temperature, and buffer composition between the reported assays.

Nevertheless, the halophilic character of R75estA may confer a distinct advantage in an industrial context. Notably, R75estA’s salinity optimum (3 M NaCl) is well above the salinity of typical marine environments (~0.5–0.6 M NaCl) and of the brackish Black Sea (~0.3 M NaCl) from which it was isolated, and at these lower salinities the enzyme retains only 8–12% of its maximal activity. Its activity instead peaks at molar-level salt concentrations, which are uncommon in natural settings but characteristic of industrial PET recycling. As discussed by Zhang et al. (2025), enzymatic PET depolymerization at high substrate loadings inevitably generates high concentrations of the acidic product TPA, which requires neutralization with NaOH. This process produces salts at molar-level concentrations, creating conditions under which conventional PETases may lose activity. R75estA is therefore most active precisely under the hypersaline conditions encountered in industrial PET processing rather than in its native marine environment, positioning it as a candidate well suited to this application. Furthermore, the emerging understanding of the structural determinants of halophilic adaptation in type III PETases, particularly the role of C-terminal acidic residues (Zhang et al., 2025), opens avenues for protein engineering strategies that could expand the operational temperature range of R75estA without compromising its salt tolerance.

## 5 Conclusion

This study reports the first biochemical characterization and structural analysis of a polyesterase from the genus *Stutzerimonas*, identified through a bioprospecting effort targeting the Western Black Sea. R75estA is the first experimentally characterized enzyme from an unstudied phylogenetic lineage within the *Pseudomonadaceae*, and one of the few type III PETases described to date, alongside PmC, HaloPETase1, and dsPETase05 (Turak et al., 2025). Analysis of the predicted structure identified several features characteristic of this recently proposed group, including the conserved pentapeptide motif, catalytic triad, disulfide bridges, substrate-binding subsites, and extended loop surrounding the active site. Functionally, R75estA hydrolyzes a broad range of substrates, from short- and long-chain esters to aliphatic and aromatic polyesters, including PET, and operates across wide temperature, pH, and salinity ranges while retaining stability in the presence of various metal ions and detergents.

The biochemical properties of R75estA suggest potential for several biotechnological applications. Its halophilic character is particularly relevant to enzymatic PET recycling, where base-mediated neutralization of the acidic product TPA generates high salt concentrations that may inactivate conventional PET hydrolases (Zhang et al., 2025). However, the limited thermophilicity of R75estA restricts its efficiency toward PET depolymerization, a limitation that may be addressed through protein engineering. Similarly, the combination of broad temperature range, alkaline pH stability, and tolerance to detergents aligns with the operational requirements of detergent formulations and textile processing, two established application domains for polyesterases (Chen et al., 2013). Taken together, these findings expand the limited sequence and functional diversity of experimentally validated type III PET hydrolases and highlight the Black Sea as an underexplored source of polyesterases with biotechnologically relevant catalytic properties.

## Data Availability

The datasets presented in this study can be found in online repositories. The names of the repository/repositories and accession number(s) can be found at: https://www.ncbi.nlm.nih.gov/genbank/, JBRACT000000000.
