# Correction: Identification and characterization of R75estA, a halophilic polyester-degrading enzyme from a marine bacterium

**DOI:** 10.3389/fmicb.2026.2028976

**Published:** 2026-10-29

**Authors:** Robert Ruginescu, Georgiana Necula-Petrareanu, Madalina Tudorache, Madalina Matei, Mihaela Muresan, Cristina Purcarea

**Affiliations:** 1 Research Institute of the University of Bucharest-ICUB, University of Bucharest, Bucharest, Romania; 2 Department of Microbiology, Institute of Biology Bucharest of the Romanian Academy, Bucharest, Romania; 3 Department of Inorganic and Organic Chemistry, Biochemistry and Catalysis, Faculty of Chemistry, University of Bucharest, Bucharest, Romania; 4 Department of Biology-Ecology, National Institute for Research and Development on Marine Geology and Geoecology-GeoEcoMar, Bucharest, Romania

**Keywords:** bioprospecting, Black Sea, cutinase, PET hydrolysis, polyesterase, *Stutzerimonas*, type III PETase

The funder European Union's Horizon 2020 research and innovation programme, grant agreement No. 101000240 (BRIDGE-BS project) was erroneously omitted. The corrected **Funding** statement reads:

“The author(s) declared that financial support was received for this work and/or its publication. This research was funded by the Romanian Academy, grant number RO1567-IBB13/2026 (to CP, RR, and GN-P); by the Research Institute of the University of Bucharest (ICUB) through the ICUB Fellowship for Young Researchers, contract number 12943/12.12.2025 (to RR); and by the European Union's Horizon 2020 research and innovation programme, BRIDGE-BS project, grant agreement No. 101000240.”

In the published article, the **Acknowledgments** were not included. The **Acknowledgments** section is below:

“The authors gratefully acknowledge the National Institute for Research and Development on Marine Geology and Geoecology – GeoEcoMar for its institutional and logistical support in the collection of seawater and sediment samples used in this study. Sampling was conducted during the MN242 research cruise aboard *R/V Mare Nigrum*, as part of the activities carried out within the Horizon 2020 BRIDGE-BS project (grant agreement No. 101000240).”

There was a mistake in the **Supplementary material** as published. The caption of **Supplementary Figure S1** and **Supplementary Table S1** did not indicate the research cruise during which the samples were collected or the original designations of the sampling stations. The corrected caption of **Supplementary Figure S1** appears below:

“**Supplementary Figure 1**. Satellite image (obtained with QGIS 3.38.3) showing the geographic locations of the nine sampling stations. Samples were collected during the MN242 research cruise of *R/V Mare Nigrum* (GeoEcoMar) within the H2020 BRIDGE-BS project. Stations are labeled with the designations used in this study (S1–S9), which correspond to the original MN242 station designations as follows: S1 = MA02, S2 = MA03, S3 = MA04, S4 = MA08, S5 = MA10, S6 = MA11, S7 = MA12, S8 = MA14, and S9 = VTZ01.”

In **Supplementary Table S1**, the original MN242 station designations have been added in parentheses next to the station designations used in this study, and the table title has been corrected to “**Supplementary Table S1**. Black Sea sampling stations (MN242 cruise of *R/V Mare Nigrum*, H2020 BRIDGE-BS project) and environmental parameters measured *in situ*.”

The corrected **Supplementary material** has been provided.

The original version of this article has been updated.

