# Supplementary material for: Identification and characterization of R75estA, a halophilic polyester-degrading enzyme from a marine bacterium
Source: Front Microbiol. 2026 Jul 10;17:1870101. doi: 10.3389/fmicb.2026.1870101 (PMC13396203; doi:10.3389/fmicb.2026.1870101)
Supplement: Supplementary file 1 [file Data_Sheet_1.PDF]

Supplementary Material

1 Supplementary Figures and Tables

1.1 Supplementary Figures

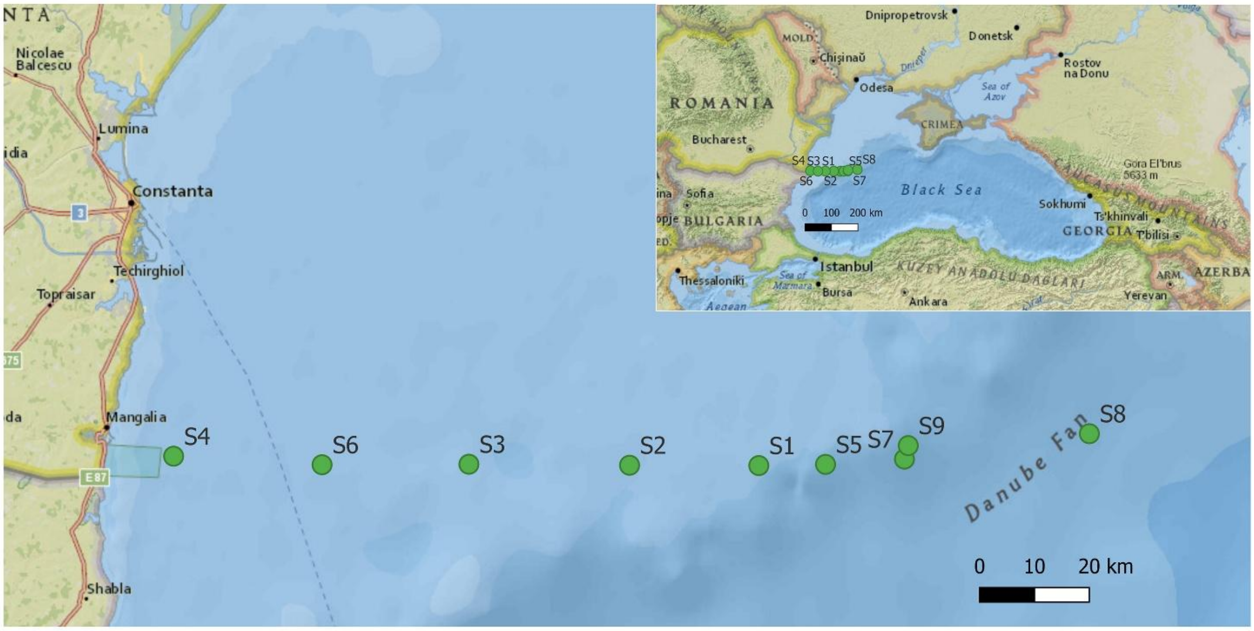

Supplementary Figure 1. Satellite image (obtained with QGIS 3.38.3) showing the geographic locations of the nine sampling stations.

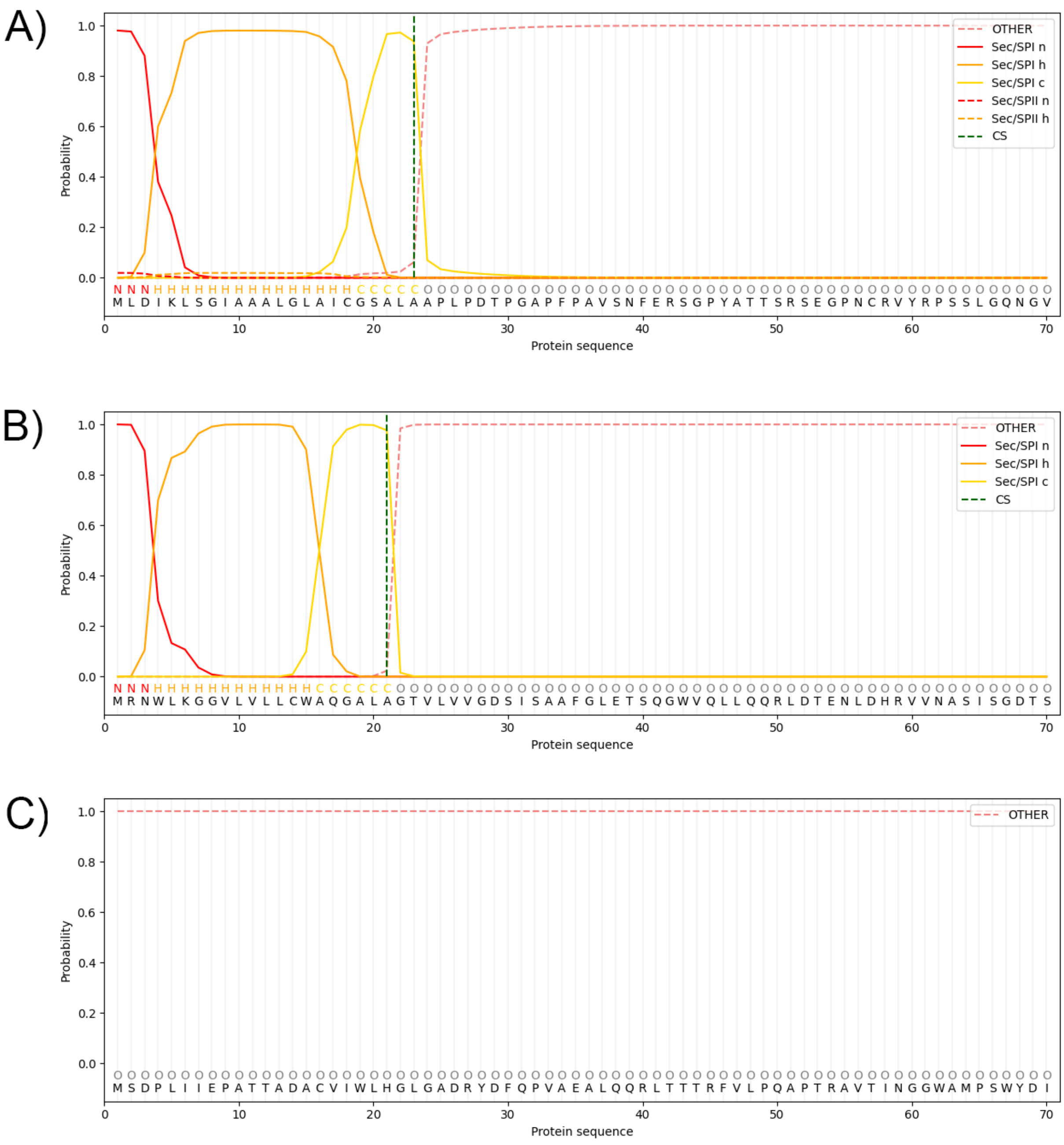

Supplementary Figure 2. SignalP 6.0 output predicting signal peptides in R75estA (**A**), R75estB (**B**), and R75estC (**C**).

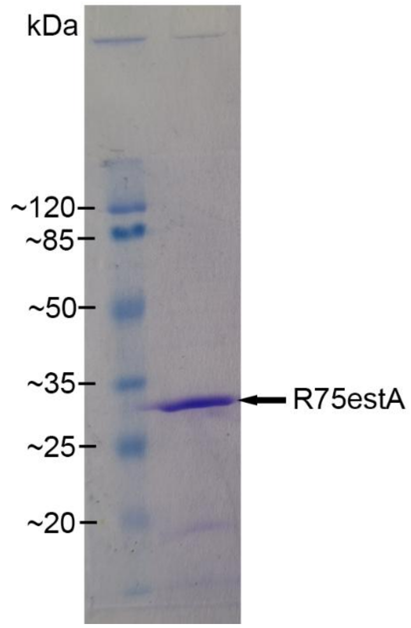

Supplementary Figure 3. SDS-PAGE analysis of purified R75estA. Lane 1: molecular weight marker; lane 2: purified R75estA.

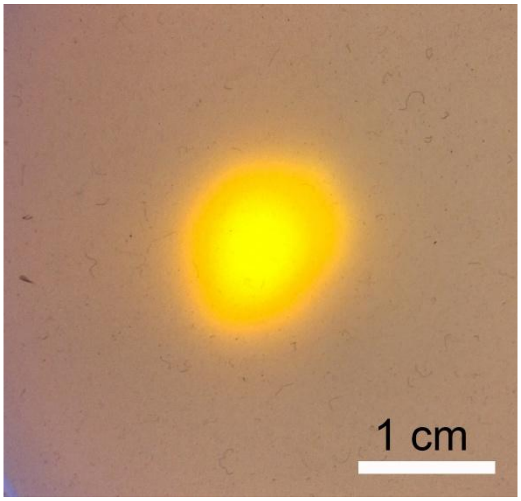

Supplementary Figure 4. Lipolytic activity of R75estA on olive oil-agar plates. The plate contains 1% (v/v) olive oil, 3.5 mg/L rhodamine B, and 17 g/L agar. Fluorescence under UV light indicates hydrolysis of olive oil by the spot-inoculated enzyme.

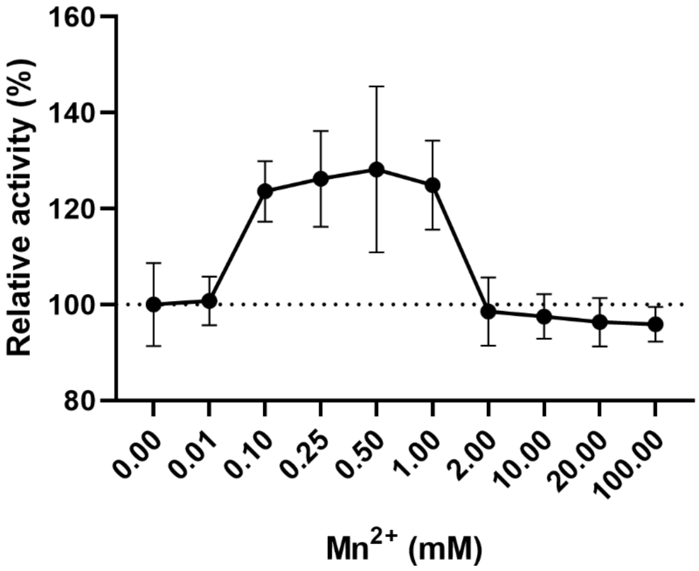

Supplementary Figure 5. Effect of Mn^{2+} concentration on R75estA activity. Relative activity of R75estA toward *p*-nitrophenyl butyrate (*p*NP-C_4) was measured in the presence of increasing Mn^{2+} concentrations (0–100 mM). Activity in the absence of Mn^{2+} was set as 100% (dotted line). Data represent the mean ± SD of three replicates.

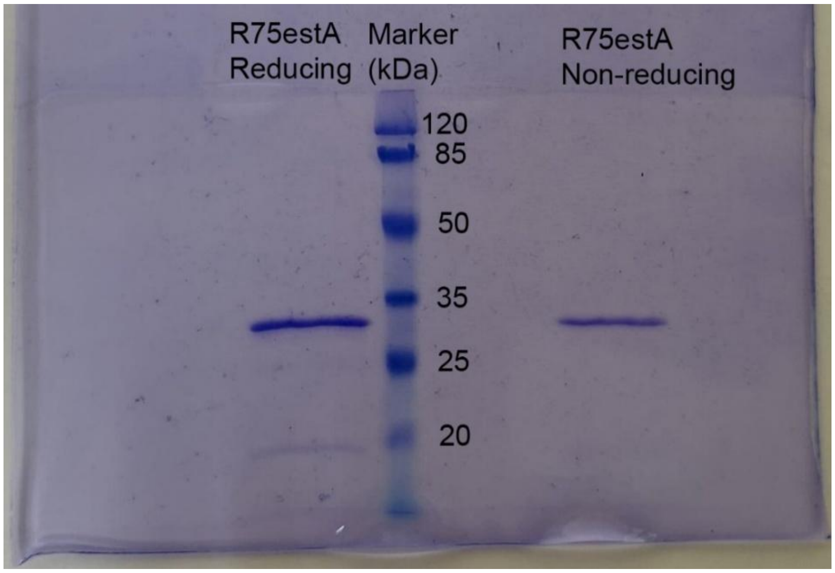

Supplementary Figure 6. SDS-PAGE analysis of purified R75estA under reducing and non-reducing conditions. Left: R75estA in the presence of β-mercaptoethanol (reducing). Center: molecular weight markers (kDa). Right: R75estA in the absence of reducing agent (non-reducing).

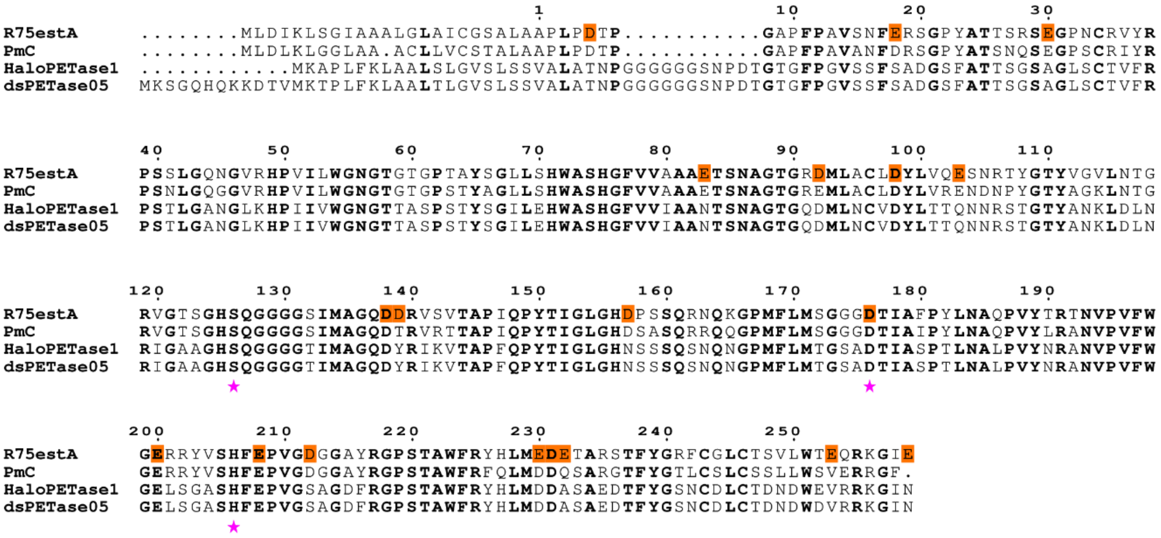

Supplementary Figure 7. Multiple sequence alignment of R75estA and homologous proteins. Catalytic triad residues are indicated by pink stars. Acidic amino acid residues (Asp and Glu) are highlighted in orange.

1.2 Supplementary Tables

Table S1. Black Sea sampling stations and environmental parameters measured *in situ*.

Station	Sample type	Depth (m)	Collection date	GPS coordinates	Temperature (°C)	pH	Salinity (PSU)	DO (mg/L)	TDS (mg/L)
S1	seawater	0.2	15.06.2023	43°45'19", 30°03'14"	19.3	7.68	16.15	9.43	17111
	sediments	114			8.6	ND	ND	ND	ND
S2	seawater	0.2	14.06.2023	43°45'21", 29°45'44"	20.1	7.66	17.69	8.88	18602
	sediments	72			8.5	ND	ND	ND	ND
S3	seawater	0.2	14.06.2023	43°45'27", 29°23'59"	19.7	7.69	17.2	9.1	18129
	sediments	68			8.6	ND	ND	ND	ND
S4	seawater	0.2	13.06.2023	43°46'14", 28°44'00"	20.9	7.43	13.08	9.24	14118
	sediments	44.8			8.6	ND	ND	ND	ND
S5	seawater	0.2	15.06.2023	43°45'26", 30°12'16"	19.6	7.69	17.92	8.87	18820
	sediments	163			8.6	ND	ND	ND	ND
S6	seawater	0.2	14.06.2023	43°45'24", 29°04'06"	20.1	7.6	15.67	8.12	16651
	sediments	57			8.6	ND	ND	ND	ND
S7	seawater	0.2	15.06.2023	43°45'57", 30°22'57"	21	7.66	17.09	8.77	18027
	sediments	476			8.6	ND	ND	ND	ND
S8	seawater	0.2	16.06.2023	43°48'26", 30°47'59"	20.7	7.57	15.09	8.91	16086
	sediments	1161			8.7	ND	ND	ND	ND
S9	seawater	20	17.06.2023	43°47'16", 30°23'28"	14.7	7.77	18.1	8.24	18995

Abbreviations: PSU (practical salinity units), DO (dissolved oxygen), TDS (total dissolved solids), ND (not determined)

Table S2. PET hydrolases used to construct the reference database.

Microbial host, enzyme designation	Isolation source	Accession no. (GenBank/UniProtKB/ PDB)
**Pseudomonadota (Proteobacteria)**		
Ideonella sakaiensis 201-F6, ISPETase	PET-contaminated samples (sediment, soil, wastewater, and activated sludge) collected at a yard of a PET bottle-recycling factory	A0A0K8P6T7
Oleispira antarctica RB-8, PET5 (=Oacut)	Antarctic coastal seawater	CCK74972.1
Vibrio gazogenes strain DSM-21264, PET6	Marine mud	WP_021018894.1
Caldimonas brevitalea, PET12 (PbCut;SbCut)	Soil sample	A0A0G3BI90_9BURK
Marinobacter sp., PLE628	Marine sediment	UUT36764.1
Marinobacter sp., PLE629	Marine sediment	UUT36763.1
Pseudomonas oleovorans/ pseudoalcaligenes, PpCutA/PoCut	Plant compost	ADK73612
Pseudomonas pelagia DSM 25163, PpelaLip	A culture of the green alga *Pyramimonas gelidicola* from the Antarctic Ocean	ANP21910.1
Halopseudomonas aestusnigri VGXO14, PE-H	Intertidal sand contaminated with crude oil	WP_088276085.1
Pseudomonas mendocina ATCC 53552, PmC	Soil sample	A4Y035
Pseudomonas saudimassiliensis, PsCut	Air samples	A0A078MGG8
Halopseudomonas bauzanensis, PbauzCut	Soil contaminated with hydrocarbon and heavy metal from an industrial site	A0A031MKR8
Pseudomonas alcaligenes, PaCut	Swimming-pool water	SUD16364.1
Moraxella sp.TA144, lip1, Mors1	Antarctic seawater	P19833
Pseudomonas pseudoalcaligenes, PpEst (tesA)	Sinus discharge	W6R2Y2
Pseudomonas sp., esterase MG8	Human saliva metagenome	NS
Pseudomonas sp. strain 9.2, EstB	Petroleum-polluted soils	WP_085690612
Rhizobacter gumimpihilus NS21, RgPETase/RgCut-I	Soil	A4W93_05950
Rhizobacter gumimpihilus, RgCut-II	Soil	WP_085749238.1
Ketobacter sp., Enzyme 403	Marine metagenome: seawater at a depth of 700 m	RLU00646.1
Ketobacter sp., Enzyme 409	Marine metagenome: seawater at a depth of 700 m	RLT92980.1
Ketobacter alkanivorans, Enzyme 412	Oil-contaminated coastal surface seawater	WP_101893509.1
Comamonadaceae bacterium SCN 68-20, Enzyme 406	Thiocyanate bioreactor metagenome	ODU60407.1
Caldimonas taiwanensis+D57, CtPL (Enzyme 504)	Hot spring	WP_062195544.1
Acidovorax delafieldii BS-3, AdCut	Soil sample	Q8RR62
Aquabacterium parvum, ApLip	Drinking water biofilms	WP_058088494.1
Burkholderiales bacterium, BurPL/Bb PETase	Soil metagenome	A0A1F4JXW8

Pseudomonas sp., GlacPETase	Glacier metagenome	NS
Halopseudomonas formosensis, Hfor_PE-H	Soil samples obtained from a composting facility	WP_090538641.1
Pseudomonas sp. JM16B3, jmPE13	Aquaculture water	NS
Pseudomonas sp. JM16B3, jmPE14	Aquaculture water	NS
Pseudomonadota bacterium, dsPETase05	North Su hydrothermal vent: 2,107m	SAMEA4473313
Pseudomonadota bacterium, dsPETase06	Seawater metagenome (Mariana Trench): 10,400m	MEC8523093.1
Brucella, PD3	Huiquan Bay (Qingdao, China)	WP_010659881.1
Pseudomonas paralcaligenes MRCP1333, PpPETase	Human feces	WP_220815207.1
Halopseudomonas lineage, HaloPETase1	Marine water sample from the Atlantic Ocean	9HL5
**Actinomycetota**		
Thermobifida fusca DSM 43793, BTA-1, TfH	Mushroom compost	Q6A0I4
T. fusca DSM 43793, BTA-2, TfH	Mushroom compost	Q6A0I3
T. fusca DSM 44342, TfH42_Cut1	Compost from green waste	E9LVI0_THEFU
T. fusca (strain YX), WSH03-11, Tfu_0883	Compost pile	Q47RJ6_THEFY
T. fusca (strain YX), WSH03-11, Tfu_0882	Compost pile	Q47RJ7_THEFY
T. fusca, TfCut_1 (Cut-1.kw3) (only active on 3PET; not on higher polymers!)	Compost	E5BBQ2
T. fusca, TfCut_2 (Cut-2.kw3)	Compost	E5BBQ3_THEFU
T. fusca NRRL B-8184, Cut1	Soil	AET05798.1
T. fusca NRRL B-8184, Cut2	Soil	AET05799.1
T. fusca, Enzyme 701	Soil	WP_104613137.1
T. fusca, Enzyme 702	Soil	ADM47605.1
T. cellulosilytica DSM44535, Thc_Cut1	Overheated manure compost	ADV92526.1
T. cellulosilytica DSM44535, Thc_Cut2	Overheated manure compost	ADV92571
T. cellulosilytica, Enzyme 711	Overheated manure compost	WP_083947829.1
T. curvata DSM43183, Tcur_1278	Straw	D1A9G5
T. curvata DSM43183, Tcur0390	Straw	ACY95991.1
T. halotolerans DSM44931*,* Thh_Est	Salt well	H6WX58
*T. alba (*AHK119)*,* Est1 (Hydrolase 4); Enzyme 708	Composted polyester films	BAI99230
T. alba DSM43185, Tha_Cut1, *cut1*	Soil	E9LVH7
T. alba AHK119, Est119, est2	Composted polyester films	F7IX06
Saccharomonospora (Thermoactinomyces) viridis AHK190, Cut190	Compost	W0TJ64
Actinobacteria *bacterium* OK074, Enzyme 405	Endosphere of plant Populus trichocarpa	WP_082414832.1
Allorhizocola rhizosphaerae, Enzyme 407	Rhizosphere soil	WP_117215036.1
Nocardioidaceae bacterium, Enzyme 503	Unknown origin (by-product of the genome assembly process during the sequencing of a genome of the fungus Coccidioides)	EGD44994.1
Marinactinospora thermotolerans, MtCut / Enzyme 606	Deep-sea sediment at about 3865 m depth	WP_078759821.1/SJZ42839.1

Actinobacteria bacterium OV320, Enzyme 607	Endosphere of plant Populus trichocarpa	WP_107095481.1
Saccharopolyspora flava, Enzyme 611	Soil	WP_093412886.1
Rhodococcus sp. RosL12, Rcut (PBATh), OL660765	Antarctic Ross Sea	UNZ22463.1
Streptomyces scabiei, Sub1	Common scab lesion on a potato tuber	QEX94755.1
Streptomyces sp. SM14, SM14est	Isolated from the sponge *Haliclona simulans* at a depth of 15 m in Kilkieran Bay, Galway, Ireland	DAC80635.1
Compost metagenome (*Actinomadura hallensis*), PHL-1	Plant waste compost (metagenome)	SAY37579.1
Compost metagenome (*Actinomadura* sp.), PHL-2	Plant waste compost (metagenome)	SAY37582.1
Amycolatopsis bacterium, PET40	Soil metagenome	WAU86704.1
Microbispora sp., SIBER-1	Soil	WOR09923
Aeromicrobium, dsPETase01	Mariana Trench: 4,000m	SAMN07748057
Dietzia kunjamensis, Dkca1	Municipal dump site, Lucknow	WID88783.1
Streptomyces calvus DSM 41452, ScPETase	Soil	QDI72884.1
**Bacillota**		
Bacillus subtilis 4P3-11m, BsEstB	Not specified	ADH43200.1
Bacillus safensis, LipMRD9	Marine sponge, South Korea: Seongsan-ri, Jeju Island	APJ12152.1
Thermoanaerobacter sp. , PHL-7 (chain B)	Plant compost	7NEI_B
Thermoanaerobacter sp. , PHL-7 (chain A)	Plant compost	7CUV_A
Compost metagenome, PHL-3[1]	Plant compost	SAY37583.1
Clostridium botulinum ATCC3502, Cbotu_EstA2	Soil	AKZ20828.1
Clostridia: Acutalibacteraceae, HGMP01	Human digestive tract	
Cryptosporangium aurantiacum, CaPETase	Lake mud	SHM40309.1
Bacillus thermoamylovorans, Lip4_120	Biofilm of PET waste in hot springs	WP_108898452.1
Bacillus thermoamylovorans, Est8_89	Biofilm of PET waste in hot springs	CEE00769.1
Bacillus thermoamylovorans, Est11_32	Biofilm of PET waste in hot springs	WP_041846030.1
Bacillus thermoamylovorans, Est18_23	Biofilm of PET waste in hot springs	WP_041847557.1
Bacillus thermoamylovorans, Ces19_14	Biofilm of PET waste in hot springs	WP_034767800.1
Bacillus thermoamylovorans, Ces39_5	Biofilm of PET waste in hot springs	WP_108898647.1
Holdemanella biformis, HG-2	Human feces	WP_118011433
Clostridiales bacterium AM23-16LB, HG-3	Human feces	RGD93181
Heyndrickxia coagulans, HG-4	Human gut	WP_013858543.1
Longicatena, HG-5	Human gut	WP_008690040.1
**Bacteroidota**		
Aequorivita sp. CIP111184, PET27	Antarctic shallow water sediments	WP_111881932.1
Kaistella (Chryseobacterium) jeonii, PET30	Moss of penguin habitats (terrestrial samples)	WP_039353427
**Chloroflexota**		
Chloroflexus sp. MS-G, Enzyme 202	Thermal spring	7QJM_A
Chloroflexi bacterium, IS10	Hydrothermal spring	UNZ81746

Chloroflexi bacterium, IS12	Hydrothermal spring	UNZ81748
Dehalococcoidia bacterium, IS11	Hydrothermal spring	UNZ81747
**Deinococcata**		
Deinococcus maricopensis DSM 21211, PET1/*Dm*PETase	Soil	E8U721
**Thermodesulfobacteriota**		
Geothermal metagenome, Sis	Hot spring samples	XDS72785.1
**Metagenome-derived without a phylogenetic affiliation**		
LCC, leaf compost metagenome, highly similar to HRB29 locus GBD22443	Leaf-branch compost metagenome	G9BY57
BhrPETase from Bacterium HR29	Subsurface geothermal stream	GBD22443
Metagenome-derived, no obvious affiliation, PET2, lipIAF5-2	Metagenome library in gelatin degradation reactor	ACC95208.1
Compost metagenome, PHL-4	Compost metagenome	SAY37584.1
Compost metagenome, PHL-5	Compost metagenome	SAY37587.1
Compost metagenome, PHL-6	Compost metagenome	SAY37589.1
**Archaea**		
Candidatus Bathyarchaeota archaeon, PET46	Deep-sea marine sediment metagenome	RLI42440.1
**Eukarya**		
Pseudozyma (Candida) antarctica, lipase B, CalB	The bottom of the antarctic lake Vanda	LIPB_PSEA2
Fusarium solani , FsC	Pea stem	AAA33335.1
Fusarium oxysporum, *Fo*Cut5a	Plants	X0BTD8
Humicola insolens, HiC	Hot spring	A0A075B5G4
Moniliophthora roreri, MRCUT1	Cacao pods	ESK97883
Dactylonectria macrodidyma, DmC	Arabidopsis root mycobiome	KAH7127633

Abbreviations: NS (not specified)

Table S3. Number of seawater isolates from nine sampling stations, grown on three different growth media.

Station	Growth media			Total
	eSWA	MG50	Marine R2A 1/10	
S1	7	6	10	23
S2	7	6	6	19
S3	15	9	7	31
S4	13	15	12	40
S5	3	4	8	15
S6	21	10	8	39
S7	5	3	6	14
S8	9	5	7	21
S9	4	1	5	10
**Total**	84	59	69	**212**

Table S4. Number of sediment isolates from eight sampling stations, grown on four different growth media.

Station	Growth media				Total
	eSWA	MG50	Marine R2A 1/10	WEM	
S1	5	4	9	3	21
S2	6	2	4	1	13
S3	7	3	2	2	14
S4	11	7	5	6	29
S5	7	4	6	0	17
S6	2	5	7	2	16
S7	16	5	6	2	29
S8	5	8	7	1	21
**Total**	59	38	46	17	**160**

Table S5. Hydrolysis of Tributyrin, Polycaprolactone Diol (PCD), and Polycaprolactone (PCL) by bacterial isolates. Presented values represent the enzymatic index (in millimeters), calculated by dividing the hydrolysis halo diameter by the diameter of the bacterial colony, after 9 days of incubation at 20 °C and an additional 14 days at 10 °C. Abbreviations: w+ (weakly positive), - (negative), ND (not determined), R (replicate), St. Dev. (standard deviation).

Isolate	Source	Station	Growth medium	Isolate publication	Tributyrin hydrolysis				PCD hydrolysis				PCL hydrolysis			
					R1	R2	Average	St. Dev.	R1	R2	Average	St. Dev.	R1	R2	Average	St. Dev.
W1	seawater	S1	eSWA	This study	1.57	1.38	1.47	0.10	ND	ND			ND	ND		
W2	seawater	S1	eSWA	This study	1.38	1.50	1.44	0.06	ND	ND			ND	ND		
W3	seawater	S1	eSWA	This study	2.56	2.00	2.28	0.28	-	-			1.75	1.64	1.69	0.06
W4	seawater	S1	eSWA	This study	2.50	2.88	2.69	0.19	2.14	2.67	2.40	0.26	-	-		
W5	seawater	S1	eSWA	This study	2.56	1.60	2.08	0.48	1.71	1.71	1.71	0.00	-	-		
W6	seawater	S1	eSWA	This study	-	-			ND	ND			ND	ND		
W7	seawater	S1	eSWA	This study	2.00	1.76	1.88	0.12	-	-			2.30	2.10	2.20	0.10
W9	seawater	S2	eSWA	This study	w+	w+			ND	ND			ND	ND		
W10	seawater	S2	eSWA	This study	2.00	2.00	2.00	0.00	-	-			-	-		
W11	seawater	S2	eSWA	This study	w+	w+			ND	ND			ND	ND		
W12	seawater	S2	eSWA	This study	2.40	2.60	2.50	0.10	-	-			1.60	1.38	1.49	0.11
W13	seawater	S2	eSWA	This study	1.77	1.77	1.77	0.00	2.15	2.36	2.26	0.10	w+	w+		
W14	seawater	S2	eSWA	This study	1.83	1.57	1.70	0.13	ND	ND			ND	ND		
W15	seawater	S2	eSWA	This study	3.27	3.50	3.39	0.11	w+	w+			1.88	1.75	1.81	0.06
W16	seawater	S3	eSWA	This study	1.80	1.80	1.80	0.00	-	-			-	-		
W17	seawater	S3	eSWA	This study	1.43	1.57	1.50	0.07	ND	ND			ND	ND		
W18	seawater	S3	eSWA	This study	1.80	2.00	1.90	0.10	-	-			-	-		
W19	seawater	S3	eSWA	This study	-	-			ND	ND			ND	ND		
W20	seawater	S3	eSWA	This study	1.89	1.50	1.69	0.19	ND	ND			ND	ND		
W21	seawater	S3	eSWA	This study	1.33	1.50	1.42	0.08	ND	ND			ND	ND		
W22	seawater	S3	eSWA	This study	1.44	1.35	1.40	0.04	ND	ND			ND	ND		
W23	seawater	S3	eSWA	This study	1.50	1.45	1.48	0.02	ND	ND			ND	ND		

W24	seawater	S3	eSWA	This study	1.64	1.80	1.72	0.08	ND	ND			ND	ND		
W25	seawater	S3	eSWA	This study	1.60	1.60	1.60	0.00	ND	ND			ND	ND		
W26	seawater	S3	eSWA	This study	2.30	2.14	2.22	0.08	-	-			1.44	1.56	1.50	0.06
W27	seawater	S3	eSWA	This study	1.80	1.80	1.80	0.00	-	-			-	-		
W28	seawater	S3	eSWA	This study	1.76	1.63	1.69	0.07	ND	ND			ND	ND		
W29	seawater	S3	eSWA	This study	1.82	1.67	1.74	0.08	-	-			-	-		
W30	seawater	S3	eSWA	This study	1.69	1.57	1.63	0.06	ND	ND			ND	ND		
W31	seawater	S4	eSWA	This study	2.18	2.10	2.14	0.04	1.56	1.63	1.59	0.03	1.63	1.43	1.53	0.10
W32	seawater	S4	eSWA	This study	1.28	1.29	1.29	0.01	ND	ND			ND	ND		
W33	seawater	S4	eSWA	This study	w+	w+			ND	ND			ND	ND		
W34	seawater	S4	eSWA	This study	w+	w+			ND	ND			ND	ND		
W35	seawater	S4	eSWA	This study	w+	w+			ND	ND			ND	ND		
W36	seawater	S5	eSWA	This study	2.56	2.67	2.61	0.06	2.33	2.40	2.37	0.03	1.50	1.56	1.53	0.03
W37	seawater	S5	eSWA	This study	w+	w+			ND	ND			ND	ND		
W38	seawater	S5	eSWA	This study	2.56	2.63	2.59	0.04	2.30	2.17	2.23	0.07	1.45	1.60	1.53	0.07
W39	seawater	S6	eSWA	This study	w+	w+			ND	ND			ND	ND		
W40	seawater	S6	eSWA	This study	w+	w+			ND	ND			ND	ND		
W41	seawater	S6	eSWA	This study	w+	w+			ND	ND			ND	ND		
W42	seawater	S6	eSWA	This study	2.09	1.93	2.01	0.08	-	-			1.45	1.40	1.43	0.03
W43	seawater	S6	eSWA	This study	1.88	1.56	1.72	0.16	ND	ND			ND	ND		
W44	seawater	S6	eSWA	This study	w+	w+			ND	ND			ND	ND		
W45	seawater	S6	eSWA	This study	w+	w+			ND	ND			ND	ND		
W46	seawater	S6	eSWA	This study	2.10	1.80	1.95	0.15	-	-			1.40	1.44	1.42	0.02
W47	seawater	S6	eSWA	This study	w+	w+			ND	ND			ND	ND		
W48	seawater	S6	eSWA	This study	2.09	1.92	2.00	0.09	-	-			1.58	1.07	1.33	0.26
W49	seawater	S6	eSWA	This study	3.60	3.17	3.38	0.22	-	-			ND	ND		
W51	seawater	S6	eSWA	This study	w+	w+			ND	ND			ND	ND		
W52	seawater	S6	eSWA	This study	2.13	2.43	2.28	0.15	-	-			2.00	2.10	2.05	0.05
W53	seawater	S6	eSWA	This study	w+	w+			ND	ND			ND	ND		
W54	seawater	S6	eSWA	This study	1.80	1.60	1.70	0.10	ND	ND			ND	ND		
W55	seawater	S7	eSWA	This study	w+	w+			ND	ND			ND	ND		

W56	seawater	S7	eSWA	This study	w+	w+			ND	ND			ND	ND		
W57	seawater	S7	eSWA	This study	2.94	3.47	3.20	0.26	-	-			1.55	1.45	1.50	0.05
W58	seawater	S7	eSWA	This study	1.60	1.50	1.55	0.05	ND	ND			ND	ND		
W59	seawater	S7	eSWA	This study	2.20	2.44	2.32	0.12	-	-			1.88	1.81	1.84	0.03
W60	seawater	S9	eSWA	This study	2.00	1.92	1.96	0.04	2.08	2.33	2.21	0.13	-	-		
W61	seawater	S9	eSWA	This study	w+	w+			ND	ND			ND	ND		
W62	seawater	S9	eSWA	This study	1.71	2.33	2.02	0.31	-	-			-	-		
W63	seawater	S9	eSWA	This study	2.19	2.18	2.19	0.00	-	-			1.63	1.56	1.59	0.03
W64	seawater	S8	eSWA	This study	w+	w+			ND	ND			ND	ND		
W65	seawater	S8	eSWA	This study	w+	w+			ND	ND			ND	ND		
W66	seawater	S8	eSWA	This study	1.84	2.10	1.97	0.13	-	-			1.50	1.44	1.47	0.03
W67	seawater	S8	eSWA	This study	2.20	2.40	2.30	0.10	w+	w+			1.53	1.47	1.50	0.03
W68	seawater	S8	eSWA	This study	w+	w+			ND	ND			ND	ND		
W69	seawater	S8	eSWA	This study	1.67	1.88	1.77	0.10	ND	ND			ND	ND		
W70	seawater	S8	eSWA	This study	w+	w+			ND	ND			ND	ND		
W71	seawater	S8	eSWA	This study	w+	w+			ND	ND			ND	ND		
W72	seawater	S4	eSWA	This study	w+	w+			ND	ND			ND	ND		
W73	seawater	S4	eSWA	This study	1.90	2.00	1.95	0.05	1.50	1.63	1.56	0.06	1.33	1.44	1.39	0.06
W74	seawater	S4	eSWA	This study	2.50	2.00	2.25	0.25	-	-			-	-		
W75	seawater	S6	eSWA	This study	w+	w+			ND	ND			ND	ND		
W76	seawater	S6	eSWA	This study	w+	w+			ND	ND			ND	ND		
W77	seawater	S6	eSWA	This study	w+	w+			ND	ND			ND	ND		
W78	seawater	S4	eSWA	This study	w+	w+			ND	ND			ND	ND		
W79	seawater	S4	eSWA	This study	w+	w+			ND	ND			ND	ND		
W80	seawater	S4	eSWA	This study	2.10	2.40	2.25	0.15	2.50	2.60	2.55	0.05	1.80	2.33	2.07	0.27
W81	seawater	S6	eSWA	This study	w+	w+			ND	ND			ND	ND		
W82	seawater	S6	eSWA	This study	1.37	w+			ND	ND			ND	ND		
W83	seawater	S6	eSWA	This study	w+	w+			ND	ND			ND	ND		
W84	seawater	S4	eSWA	This study	2.23	2.50	2.37	0.13	w+	w+			-	-		
W85	seawater	S4	eSWA	This study	1.58	1.60	1.59	0.01	1.86	2.14	2.00	0.14	w+	w+		
W86	seawater	S8	eSWA	This study	1.57	1.43	1.50	0.07	ND	ND			ND	ND		

WS1	sediments	S2	eSWA	This study	2.10	2.00	2.05	0.05	-	-			-	-		
WS2	sediments	S2	eSWA	This study	1.70	1.70	1.70	0.00	-	-			-	-		
WS3	sediments	S2	eSWA	This study	1.90	2.11	2.01	0.11	-	-			-	-		
WS4	sediments	S2	eSWA	This study	w+	w+			ND	ND			ND	ND		
WS5	sediments	S2	eSWA	This study	1.65	1.50	1.57	0.07	ND	ND			ND	ND		
WS6	sediments	S2	eSWA	This study	w+	w+			ND	ND			ND	ND		
WS7	sediments	S1	eSWA	This study	w+	w+			ND	ND			ND	ND		
WS8	sediments	S1	eSWA	This study	1.67	1.65	1.66	0.01	ND	ND			ND	ND		
WS9	sediments	S1	eSWA	This study	1.75	1.75	1.75	0.00	ND	ND			ND	ND		
WS10	sediments	S1	eSWA	This study	1.88	1.67	1.77	0.10	ND	ND			ND	ND		
WS11	sediments	S3	eSWA	This study	1.50	1.50	1.50	0.00	ND	ND			ND	ND		
WS12	sediments	S3	eSWA	This study	1.67	1.88	1.77	0.10	ND	ND			ND	ND		
WS13	sediments	S3	eSWA	This study	w+	w+			ND	ND			ND	ND		
WS15	sediments	S3	eSWA	This study	w+	w+			ND	ND			ND	ND		
WS16	sediments	S3	eSWA	This study	1.60	1.60	1.60	0.00	ND	ND			ND	ND		
WS17	sediments	S3	eSWA	This study	3.14	3.13	3.13	0.01	-	-			2.53	2.31	2.42	0.11
WS18	sediments	S4	eSWA	This study	1.56	1.75	1.65	0.10	ND	ND			ND	ND		
WS20	sediments	S4	eSWA	This study	1.63	2.00	1.81	0.19	-	-			-	-		
WS21	sediments	S4	eSWA	This study	1.52	2.86	2.19	0.67	-	-			-	-		
WS22	sediments	S4	eSWA	This study	1.44	1.44	1.44	0.00	ND	ND			ND	ND		
WS23	sediments	S4	eSWA	This study	1.50	1.75	1.63	0.13	ND	ND			ND	ND		
WS24	sediments	S5	eSWA	This study	1.60	1.60	1.60	0.00	ND	ND			ND	ND		
WS28	sediments	S5	eSWA	This study	w+	w+			ND	ND			ND	ND		
WS29	sediments	S5	eSWA	This study	2.00	2.00	2.00	0.00	-	-			-	-		
WS30	sediments	S5	eSWA	This study	1.67	2.60	2.13	0.47	ND	ND			ND	ND		
WS31	sediments	S5	eSWA	This study	1.50	1.56	1.53	0.03	ND	ND			ND	ND		
WS32	sediments	S5	eSWA	This study	2.00	1.80	1.90	0.10	-	-			1.78	1.45	1.62	0.16
WS33	sediments	S5	eSWA	This study	1.40	1.60	1.50	0.10	ND	ND			ND	ND		
WS38	sediments	S6	eSWA	This study	w+	w+			ND	ND			ND	ND		
WS39	sediments	S6	eSWA	This study	2.50	1.53	2.02	0.48	-	-			-	-		
WS40	sediments	S8	eSWA	This study	-	-			ND	ND			ND	ND		

WS41	sediments	S8	eSWA	This study	1.88	1.56	1.72	0.16	ND	ND			ND	ND		
WS42	sediments	S8	eSWA	This study	1.78	1.79	1.78	0.00	2.86	2.86	2.86	0.00	1.50	1.47	1.49	0.01
WS43	sediments	S8	eSWA	This study	1.94	2.13	2.03	0.09	-	-			-	-		
WS44	sediments	S8	eSWA	This study	2.00	1.83	1.92	0.08	-	-			-	-		
WS45	sediments	S7	eSWA	This study	-	-			ND	ND			ND	ND		
WS46	sediments	S7	eSWA	This study	1.54	1.67	1.60	0.06	ND	ND			ND	ND		
WS47	sediments	S7	eSWA	This study	1.69	1.83	1.76	0.07	ND	ND			ND	ND		
WS48	sediments	S7	eSWA	This study	w+	w+			ND	ND			ND	ND		
WS49	sediments	S7	eSWA	This study	-	-			ND	ND			ND	ND		
WS51	sediments	S7	eSWA	This study	w+	w+			ND	ND			ND	ND		
WS52	sediments	S7	eSWA	This study	w+	w+			ND	ND			ND	ND		
WS54	sediments	S7	eSWA	This study	2.38	2.31	2.34	0.03	-	-			-	-		
WS55	sediments	S7	eSWA	This study	2.63	2.56	2.59	0.03	2.17	2.17	2.17	0.00	3.71	3.25	3.48	0.23
WS57	sediments	S7	eSWA	This study	w+	w+			ND	ND			ND	ND		
WS58	sediments	S7	eSWA	This study	-	-			ND	ND			ND	ND		
WS59	sediments	S7	eSWA	This study	-	-			ND	ND			ND	ND		
WS60	sediments	S7	eSWA	This study	-	-			ND	ND			ND	ND		
WS61	sediments	S7	eSWA	This study	w+	w+			ND	ND			ND	ND		
WS62	sediments	S7	eSWA	This study	1.39	1.64	1.52	0.13	2.00	2.08	2.04	0.04	4.29	3.29	3.79	0.50
WS64	sediments	S4	eSWA	This study	-	-			ND	ND			ND	ND		
WS65	sediments	S4	eSWA	This study	w+	w+			ND	ND			ND	ND		
WS66	sediments	S4	eSWA	This study	w+	w+			ND	ND			ND	ND		
WS67	sediments	S4	eSWA	This study	w+	w+			ND	ND			ND	ND		
WS68	sediments	S1	eSWA	This study	w+	w+			ND	ND			ND	ND		
WS69	sediments	S3	eSWA	This study	w+	w+			ND	ND			ND	ND		
WS70	sediments	S4	eSWA	This study	-	-			ND	ND			ND	ND		
WS71	sediments	S7	eSWA	This study	1.41	1.29	1.35	0.06	ND	ND			ND	ND		
WS72	sediments	S4	eSWA	This study	w+	w+			ND	ND			ND	ND		
G1	seawater	S1	MG50	This study	w+	w+			ND	ND			ND	ND		
G2	seawater	S1	MG50	This study	2.70	2.70	2.70	0.00	1.65	1.56	1.60	0.05	2.23	2.75	2.49	0.26
G4	seawater	S1	MG50	This study	1.90	1.64	1.77	0.13	ND	ND			ND	ND		

G5	seawater	S1	MG50	This study	w+	w+			ND	ND			ND	ND		
G6	seawater	S1	MG50	This study	1.78	1.60	1.69	0.09	ND	ND			ND	ND		
G7	seawater	S1	MG50	This study	1.50	1.67	1.58	0.08	ND	ND			ND	ND		
G8	seawater	S2	MG50	This study	w+	w+			ND	ND			ND	ND		
G9	seawater	S2	MG50	This study	1.80	1.80	1.80	0.00	-	-			-	-		
G10	seawater	S2	MG50	This study	1.50	1.50	1.50	0.00	ND	ND			ND	ND		
G11	seawater	S2	MG50	This study	1.29	1.43	1.36	0.07	ND	ND			ND	ND		
G12	seawater	S2	MG50	This study	1.54	1.43	1.48	0.05	ND	ND			ND	ND		
G13	seawater	S2	MG50	This study	1.47	1.30	1.39	0.09	ND	ND			ND	ND		
G14	seawater	S3	MG50	This study	1.80	1.80	1.80	0.00	-	-			-	-		
G16	seawater	S3	MG50	This study	1.44	1.40	1.42	0.02	ND	ND			ND	ND		
G17	seawater	S3	MG50	This study	1.80	2.00	1.90	0.10	-	-			-	-		
G18	seawater	S3	MG50	This study	w+	w+			ND	ND			ND	ND		
G19	seawater	S3	MG50	This study	2.20	2.00	2.10	0.10	-	-			1.56	1.56	1.56	0.00
G20	seawater	S3	MG50	This study	w+	w+			ND	ND			ND	ND		
G21	seawater	S3	MG50	This study	w+	w+			ND	ND			ND	ND		
G22	seawater	S3	MG50	This study	2.00	2.00	2.00	0.00	-	-			-	-		
G23	seawater	S4	MG50	This study	1.80	1.60	1.70	0.10	ND	ND			ND	ND		
G25	seawater	S4	MG50	This study	w+	w+			ND	ND			ND	ND		
G27	seawater	S4	MG50	This study	w+	w+			ND	ND			ND	ND		
G28	seawater	S4	MG50	This study	w+	w+			ND	ND			ND	ND		
G29	seawater	S4	MG50	This study	1.67	w+	1.67		ND	ND			ND	ND		
G31	seawater	S4	MG50	This study	2.15	2.40	2.28	0.13	1.40	1.33	1.37	0.03	w+	w+		
G32	seawater	S4	MG50	This study	1.60	1.50	1.55	0.05	-	-			-	-		
G34	seawater	S4	MG50	This study	w+	w+			ND	ND			ND	ND		
G35	seawater	S4	MG50	This study	2.67	2.50	2.58	0.08	-	-			-	-		
G36	seawater	S4	MG50	This study	w+	w+			ND	ND			ND	ND		
G38	seawater	S4	MG50	This study	w+	w+			ND	ND			ND	ND		
G39	seawater	S4	MG50	This study	w+	w+			ND	ND			ND	ND		
G40	seawater	S4	MG50	This study	w+	w+			ND	ND			ND	ND		
G41	seawater	S4	MG50	This study	w+	w+			ND	ND			ND	ND		

G43	seawater	S6	MG50	This study	w+	w+			ND	ND			ND	ND		
G44	seawater	S6	MG50	This study	1.67	1.63	1.65	0.02	ND	ND			ND	ND		
G45	seawater	S6	MG50	This study	1.63	1.63	1.63	0.00	ND	ND			ND	ND		
G46	seawater	S6	MG50	This study	1.69	1.39	1.54	0.15	ND	ND			ND	ND		
G47	seawater	S6	MG50	This study	1.44	1.25	1.34	0.09	-	-			-	-		
G48	seawater	S6	MG50	This study	w+	w+			ND	ND			ND	ND		
G49	seawater	S6	MG50	This study	w+	w+			ND	ND			ND	ND		
G50	seawater	S6	MG50	This study	w+	w+			ND	ND			ND	ND		
G51	seawater	P9	MG50	This study	w+	w+			ND	ND			ND	ND		
G53	seawater	P9	MG50	This study	w+	w+			ND	ND			ND	ND		
G54	seawater	P9	MG50	This study	2.00	1.83	1.92	0.08	w+	w+			1.75	1.75	1.75	0.00
G55	seawater	S8	MG50	This study	w+	w+			ND	ND			ND	ND		
G56	seawater	S8	MG50	This study	w+	w+			ND	ND			ND	ND		
G57	seawater	S8	MG50	This study	w+	w+			ND	ND			ND	ND		
G58	seawater	S8	MG50	This study	-	-			ND	ND			ND	ND		
G59	seawater	S8	MG50	This study	1.75	1.60	1.68	0.08	ND	ND			ND	ND		
G61	seawater	S5	MG50	This study	1.67	2.00	1.83	0.17	2.23	2.50	2.37	0.13	-	-		
G62	seawater	S5	MG50	This study	w+	w+			ND	ND			ND	ND		
G64	seawater	S5	MG50	This study	1.50	1.60	1.55	0.05	ND	ND			ND	ND		
G65	seawater	S5	MG50	This study	w+	w+			ND	ND			ND	ND		
G66	seawater	S4	MG50	This study	1.58	1.82	1.70	0.12	ND	ND			ND	ND		
G67	seawater	S9	MG50	This study	1.60	1.31	1.45	0.15	ND	ND			ND	ND		
G68	seawater	S6	MG50	This study	-	-			ND	ND			ND	ND		
G69	seawater	S3	MG50	This study	2.20	1.60	1.90	0.30	-	-			-	-		
G70	seawater	S6	MG50	This study	w+	w+			ND	ND			ND	ND		
GS1	sediments	S1	MG50	This study	-	-			ND	ND			ND	ND		
GS2	sediments	S1	MG50	This study	-	-			ND	ND			ND	ND		
GS3	sediments	S1	MG50	This study	2.38	2.00	2.19	0.19	-	-			-	-		
GS5	sediments	S1	MG50	This study	w+	w+			ND	ND			ND	ND		
GS7	sediments	S2	MG50	This study	1.89	1.83	1.86	0.03	-	-			-	-		
GS8	sediments	S2	MG50	This study	-	-			ND	ND			ND	ND		

GS9	sediments	S3	MG50	This study	w+	w+			ND	ND			ND	ND		
GS10	sediments	S3	MG50	This study	w+	w+			ND	ND			ND	ND		
GS11	sediments	S4	MG50	This study	w+	w+			ND	ND			ND	ND		
GS12	sediments	S4	MG50	This study	w+	w+			ND	ND			ND	ND		
GS13	sediments	S4	MG50	This study	2.00	1.53	1.77	0.23	ND	ND			ND	ND		
GS14	sediments	S4	MG50	This study	1.56	1.61	1.58	0.03	ND	ND			ND	ND		
GS15	sediments	S4	MG50	This study	1.88	2.00	1.94	0.06	w+	w+			2.85	3.00	2.93	0.07
GS16	sediments	S4	MG50	This study	2.00	1.50	1.75	0.25	ND	ND			ND	ND		
GS17	sediments	S5	MG50	This study	w+	w+			ND	ND			ND	ND		
GS18	sediments	S5	MG50	This study	w+	w+			ND	ND			ND	ND		
GS19	sediments	S5	MG50	This study	1.50	1.38	1.44	0.06	ND	ND			ND	ND		
GS20	sediments	S5	MG50	This study	w+	w+			ND	ND			ND	ND		
GS21	sediments	S6	MG50	This study	2.00	2.40	2.20	0.20	-	-			-	-		
GS23	sediments	S6	MG50	This study	1.73	1.75	1.74	0.01	ND	ND			ND	ND		
GS24	sediments	S6	MG50	This study	w+	w+			ND	ND			ND	ND		
GS25	sediments	S6	MG50	This study	1.90	1.90	1.90	0.00	-	-			-	-		
GS26	sediments	S6	MG50	This study	2.00	2.00	2.00	0.00	-	-			4.29	4.43	4.36	0.07
GS28	sediments	S7	MG50	This study	w+	w+			ND	ND			ND	ND		
GS29	sediments	S7	MG50	This study	1.89	1.80	1.85	0.05	-	-			-	-		
GS30	sediments	S7	MG50	This study	w+	w+			ND	ND			ND	ND		
GS31	sediments	S8	MG50	This study	2.13	1.78	1.95	0.17	-	-			-	-		
GS32	sediments	S8	MG50	This study	w+	w+			ND	ND			ND	ND		
GS33	sediments	S8	MG50	This study	2.63	1.67	2.15	0.48	1.85	1.92	1.88	0.04	w+	w+		
GS34	sediments	S8	MG50	This study	-	-			ND	ND			ND	ND		
GS35	sediments	S8	MG50	This study	1.43	1.54	1.48	0.05	ND	ND			ND	ND		
GS36	sediments	S8	MG50	This study	2.11	1.60	1.86	0.26	-	-			-	-		
GS37	sediments	S8	MG50	This study	1.54	1.33	1.44	0.10	ND	ND			ND	ND		
GS38	sediments	S8	MG50	This study	2.20	1.57	1.89	0.31	-	-			-	-		
GS39	sediments	S4	MG50	This study	1.60	2.31	1.95	0.35	-	-			-	-		
GS41	sediments	S7	MG50	This study	w+	w+			ND	ND			ND	ND		
GS42	sediments	S3	MG50	This study	1.41	1.50	1.45	0.05	ND	ND			ND	ND		

GS43	sediments	S7	MG50	This study	w+	w+			ND	ND			ND	ND		
R1	seawater	S1	Marine R2A 1/10	This study	2.00	1.93	1.96	0.04	-	-			-	-		
R2	seawater	S1	Marine R2A 1/10	This study	1.90	2.11	2.00	0.10	2.91	2.91	2.91	0.00	w+	w+		
R3	seawater	S1	Marine R2A 1/10	This study	2.50	2.18	2.34	0.16	3.00	2.88	2.94	0.06	-	-		
R4	seawater	S1	Marine R2A 1/10	This study	1.71	1.60	1.66	0.06	-	-			-	-		
R5	seawater	S1	Marine R2A 1/10	This study	1.48	1.56	1.52	0.04	ND	ND			ND	ND		
R7	seawater	S1	Marine R2A 1/10	This study	1.28	w+			ND	ND			ND	ND		
R8	seawater	S1	Marine R2A 1/10	This study	1.44	1.50	1.47	0.03	ND	ND			ND	ND		
R9	seawater	S1	Marine R2A 1/10	This study	1.90	1.90	1.90	0.00	-	-			1.36	1.50	1.43	0.07
R10	seawater	S1	Marine R2A 1/10	This study	1.27	1.50	1.39	0.11	ND	ND			ND	ND		
R11	seawater	S1	Marine R2A 1/10	This study	1.50	1.38	1.44	0.06	ND	ND			ND	ND		
R12	seawater	S2	Marine R2A 1/10	This study	w+	w+			ND	ND			ND	ND		
R15	seawater	S2	Marine R2A 1/10	This study	w+	w+			ND	ND			ND	ND		
R18	seawater	S2	Marine R2A 1/10	This study	2.00	2.60	2.30	0.30	1.58	1.90	1.74	0.16	2.15	2.00	2.08	0.08

R21	seawater	S2	Marine R2A 1/10	This study	1.54	1.43	1.48	0.05	-	-			-	-		
R24	seawater	S2	Marine R2A 1/10	This study	w+	w+			ND	ND			ND	ND		
R25	seawater	S2	Marine R2A 1/10	This study	w+	w+			ND	ND			ND	ND		
R27	seawater	S3	Marine R2A 1/10	This study	1.78	1.60	1.69	0.09	ND	ND			ND	ND		
R28	seawater	S3	Marine R2A 1/10	This study	2.13	1.88	2.00	0.13	-	-			-	-		
R31	seawater	S3	Marine R2A 1/10	This study	w+	w+			ND	ND			ND	ND		
R32	seawater	S3	Marine R2A 1/10	This study	1.60	1.60	1.60	0.00	ND	ND			ND	ND		
R33	seawater	S3	Marine R2A 1/10	This study	1.45	1.60	1.53	0.07	ND	ND			ND	ND		
R34	seawater	S3	Marine R2A 1/10	This study	1.76	1.88	1.82	0.06	-	-			-	-		
R35	seawater	S3	Marine R2A 1/10	This study	-	-			ND	ND			ND	ND		
R36	seawater	S4	Marine R2A 1/10	This study	w+	w+			ND	ND			ND	ND		
R38	seawater	S4	Marine R2A 1/10	This study	2.19	1.81	2.00	0.19	-	-			w+	w+		
R39	seawater	S4	Marine R2A 1/10	This study	w+	w+			ND	ND			ND	ND		

R41	seawater	S4	Marine R2A 1/10	This study	w+	w+			ND	ND			ND	ND		
R42	seawater	S4	Marine R2A 1/10	This study	w+	w+			ND	ND			ND	ND		
R43	seawater	S4	Marine R2A 1/10	This study	w+	w+			ND	ND			ND	ND		
R44	seawater	S4	Marine R2A 1/10	This study	w+	w+			ND	ND			ND	ND		
R48	seawater	S4	Marine R2A 1/10	This study	w+	w+			ND	ND			ND	ND		
R52	seawater	S6	Marine R2A 1/10	This study	1.90	2.00	1.95	0.05	2.36	2.29	2.32	0.04	w+	w+		
R53	seawater	S7	Marine R2A 1/10	This study	w+	w+			ND	ND			ND	ND		
R55	seawater	S7	Marine R2A 1/10	This study	1.57	1.57	1.57	0.00	-	-			-	-		
R57	seawater	S7	Marine R2A 1/10	This study	2.10	2.11	2.10	0.00	ND	ND			ND	ND		
R59	seawater	S7	Marine R2A 1/10	This study	1.78	2.22	2.00	0.22	2.15	2.33	2.24	0.09	-	-		
R60	seawater	S7	Marine R2A 1/10	This study	1.70	1.80	1.75	0.05	ND	ND			ND	ND		
R61	seawater	S7	Marine R2A 1/10	This study	1.75	1.82	1.78	0.03	ND	ND			ND	ND		
R62	seawater	S5	Marine R2A 1/10	This study	1.71	2.00	1.86	0.14	w+	w+			-	-		

R63	seawater	S5	Marine R2A 1/10	This study	1.67	2.00	1.83	0.17	-	-			w+	w+		
R64	seawater	S5	Marine R2A 1/10	This study	2.22	1.65	1.94	0.29	-	-			1.69	1.32	1.51	0.19
R65	seawater	S5	Marine R2A 1/10	This study	1.86	2.00	1.93	0.07	-	-			w+	w+		
R66	seawater	S5	Marine R2A 1/10	This study	1.80	1.87	1.83	0.03	-	-			w+	w+		
R67	seawater	S5	Marine R2A 1/10	This study	1.47	1.47	1.47	0.00	ND	ND			ND	ND		
R68	seawater	S5	Marine R2A 1/10	This study	1.67	2.10	1.88	0.22	-	-			w+	w+		
R69	seawater	S5	Marine R2A 1/10	This study	w+	w+			ND	ND			ND	ND		
R70	seawater	S8	Marine R2A 1/10	This study	-	-			ND	ND			ND	ND		
R72	seawater	S8	Marine R2A 1/10	This study	1.41	1.44	1.42	0.01	ND	ND			ND	ND		
R73	seawater	S8	Marine R2A 1/10	This study	w+	w+			ND	ND			ND	ND		
R74	seawater	S8	Marine R2A 1/10	This study	2.38	2.00	2.19	0.19	w+	w+			1.39	1.47	1.43	0.04
R75	seawater	S8	Marine R2A 1/10	This study	2.75	2.00	2.38	0.38	3.00	3.00	3.00	0.00	2.62	2.13	2.37	0.24
R76	seawater	S8	Marine R2A 1/10	This study	-	-			ND	ND			ND	ND		

R77	seawater	S8	Marine R2A 1/10	This study	w+	w+			ND	ND			ND	ND		
R79	seawater	S9	Marine R2A 1/10	This study	2.00	1.67	1.83	0.17	-	-			-	-		
R80	seawater	S9	Marine R2A 1/10	This study	-	-			ND	ND			ND	ND		
R81	seawater	S9	Marine R2A 1/10	This study	1.82	1.67	1.74	0.08	ND	ND			ND	ND		
R82	seawater	S9	Marine R2A 1/10	This study	1.71	1.92	1.82	0.10	-	-			-	-		
R83	seawater	S9	Marine R2A 1/10	This study	2.30	2.30	2.30	0.00	1.85	1.86	1.85	0.01	1.63	1.88	1.75	0.13
R85	seawater	S4	Marine R2A 1/10	This study	w+	w+			ND	ND			ND	ND		
R87	seawater	S4	Marine R2A 1/10	This study	w+	w+			ND	ND			ND	ND		
R88	seawater	S4	Marine R2A 1/10	This study	3.00	2.60	2.80	0.20	1.82	1.82	1.82	0.00	1.73	2.10	1.91	0.19
R89	seawater	S4	Marine R2A 1/10	This study	2.00	1.70	1.85	0.15	-	-			1.69	1.57	1.63	0.06
R90	seawater	S6	Marine R2A 1/10	This study	1.54	1.43	1.48	0.05	ND	ND			ND	ND		
R91	seawater	S6	Marine R2A 1/10	This study	-	-			ND	ND			ND	ND		
R93	seawater	S6	Marine R2A 1/10	This study	1.45	1.45	1.45	0.00	ND	ND			ND	ND		

R94	seawater	S6	Marine R2A 1/10	This study	1.60	1.36	1.48	0.12	ND	ND			ND	ND		
R95	seawater	S6	Marine R2A 1/10	This study	2.55	2.36	2.45	0.09	-	-			-	-		
R96	seawater	S6	Marine R2A 1/10	This study	w+	w+			ND	ND			ND	ND		
R97	seawater	S6	Marine R2A 1/10	This study	1.33	1.43	1.38	0.05	ND	ND			ND	ND		
RS3	sediments	S2	Marine R2A 1/10	This study	w+	w+			ND	ND			ND	ND		
RS4	sediments	S2	Marine R2A 1/10	This study	1.52	1.45	1.49	0.03	ND	ND			ND	ND		
RS8	sediments	S2	Marine R2A 1/10	This study	1.82	1.82	1.82	0.00	-	-			-	-		
RS9	sediments	S2	Marine R2A 1/10	This study	1.42	1.45	1.44	0.02	ND	ND			ND	ND		
RS11	sediments	S7	Marine R2A 1/10	This study	w+	w+			ND	ND			ND	ND		
RS12	sediments	S7	Marine R2A 1/10	This study	-	-			ND	ND			ND	ND		
RS13	sediments	S7	Marine R2A 1/10	This study	w+	w+			ND	ND			ND	ND		
RS14	sediments	S7	Marine R2A 1/10	This study	w+	w+			ND	ND			ND	ND		
RS15	sediments	S7	Marine R2A 1/10	This study	-	-			ND	ND			ND	ND		

RS16	sediments	S7	Marine R2A 1/10	This study	w+	w+			ND	ND			ND	ND		
RS17	sediments	S8	Marine R2A 1/10	This study	-	-			ND	ND			ND	ND		
RS18	sediments	S8	Marine R2A 1/10	This study	w+	w+			ND	ND			ND	ND		
RS19	sediments	S8	Marine R2A 1/10	This study	w+	w+			ND	ND			ND	ND		
RS20	sediments	S8	Marine R2A 1/10	This study	w+	w+			ND	ND			ND	ND		
RS21	sediments	S8	Marine R2A 1/10	This study	2.50	2.71	2.60	0.10	3.93	3.85	3.89	0.04	1.33	1.39	1.36	0.03
RS22	sediments	S8	Marine R2A 1/10	This study	-	-			ND	ND			ND	ND		
RS23	sediments	S8	Marine R2A 1/10	This study	1.53	1.65	1.59	0.06	ND	ND			ND	ND		
RS29	sediments	S5	Marine R2A 1/10	This study	w+	w+			ND	ND			ND	ND		
RS30	sediments	S5	Marine R2A 1/10	This study	1.57	1.22	1.40	0.17	ND	ND			ND	ND		
RS32	sediments	S5	Marine R2A 1/10	This study	-	-			ND	ND			ND	ND		
RS33	sediments	S5	Marine R2A 1/10	This study	-	-			ND	ND			ND	ND		
RS34	sediments	S5	Marine R2A 1/10	This study	w+	w+			ND	ND			ND	ND		

RS35	sediments	S5	Marine R2A 1/10	This study	w+	w+			ND	ND			ND	ND		
RS36	sediments	S1	Marine R2A 1/10	This study	w+	w+			ND	ND			ND	ND		
RS38	sediments	S1	Marine R2A 1/10	This study	w+	w+			ND	ND			ND	ND		
RS39	sediments	S1	Marine R2A 1/10	This study	1.90	1.76	1.83	0.07	-	-			-	-		
RS40	sediments	S1	Marine R2A 1/10	This study	2.00	2.00	2.00	0.00	2.00	1.92	1.96	0.04	w+	w+		
RS41	sediments	S1	Marine R2A 1/10	This study	w+	w+			ND	ND			ND	ND		
RS43	sediments	S1	Marine R2A 1/10	This study	2.80	2.27	2.54	0.26	-	-			-	-		
RS44	sediments	S1	Marine R2A 1/10	This study	2.20	1.75	1.98	0.23	-	-			-	-		
RS45	sediments	S1	Marine R2A 1/10	This study	w+	w+			ND	ND			ND	ND		
RS46	sediments	S1	Marine R2A 1/10	This study	1.60	1.80	1.70	0.10	ND	ND			ND	ND		
RS50	sediments	S3	Marine R2A 1/10	This study	w+	w+			ND	ND			ND	ND		
RS52	sediments	S3	Marine R2A 1/10	This study	w+	w+			ND	ND			ND	ND		
RS58	sediments	S6	Marine R2A 1/10	This study	1.50	1.50	1.50	0.00	ND	ND			ND	ND		

RS59	sediments	S6	Marine R2A 1/10	This study	w+	w+			ND	ND			ND	ND		
RS61	sediments	S6	Marine R2A 1/10	This study	w+	w+			ND	ND			ND	ND		
RS62	sediments	S6	Marine R2A 1/10	This study	-	-			ND	ND			ND	ND		
RS63	sediments	S6	Marine R2A 1/10	This study	w+	w+			ND	ND			ND	ND		
RS64	sediments	S6	Marine R2A 1/10	This study	w+	w+			ND	ND			ND	ND		
RS65	sediments	S6	Marine R2A 1/10	This study	w+	w+			ND	ND			ND	ND		
RS67	sediments	S4	Marine R2A 1/10	This study	1.70	1.40	1.55	0.15	ND	ND			ND	ND		
RS71	sediments	S4	Marine R2A 1/10	This study	w+	w+			ND	ND			ND	ND		
RS73	sediments	S4	Marine R2A 1/10	This study	w+	w+			ND	ND			ND	ND		
RS79	sediments	S4	Marine R2A 1/10	This study	1.43	1.36			ND	ND			ND	ND		
RS80	sediments	S4	Marine R2A 1/10	This study	1.63	1.63			ND	ND			ND	ND		
WEM1	sediments	S1	WEM	This study	w+	w+			ND	ND			ND	ND		
WEM2	sediments	S1	WEM	This study	1.80	2.00	1.90	0.10	-	-			2.89	3.00	2.94	0.06
WEM3	sediments	S1	WEM	This study	1.45	1.45	1.45	0.00	ND	ND			ND	ND		
WEM4	sediments	S2	WEM	This study	1.56	1.75	1.66	0.09	ND	ND			ND	ND		
WEM5	sediments	S3	WEM	This study	w+	w+			ND	ND			ND	ND		
WEM6	sediments	S7	WEM	This study	2.11	1.67	1.89	0.22	-	-			-	-		

WEM7	sediments	S7	WEM	This study	w+	w+			ND	ND			ND	ND		
WEM8	sediments	S8	WEM	This study	-	-			ND	ND			ND	ND		
WEM10	sediments	S4	WEM	This study	1.39	1.18	1.28	0.11	ND	ND			ND	ND		
WEM11	sediments	S4	WEM	This study	w+	w+			ND	ND			ND	ND		
WEM12	sediments	S4	WEM	This study	w+	w+			ND	ND			ND	ND		
WEM13	sediments	S4	WEM	This study	w+	w+			ND	ND			ND	ND		
WEM14	sediments	S6	WEM	This study	w+	w+			ND	ND			ND	ND		
WEM16	sediments	S6	WEM	This study	w+	w+			ND	ND			ND	ND		
WEM17	sediments	S4	WEM	This study	w+	w+			ND	ND			ND	ND		
WEM19	sediments	S3	WEM	This study	w+	w+			ND	ND			ND	ND		
WEM21	sediments	S4	WEM	This study	w+	w+			ND	ND			ND	ND		
SWA CA P1.19	seawater	CA	eSWA	Ruginescu et al., 2022	1.30	1.30	1.30	0.00	ND	ND			ND	ND		
MA CA P2.3	seawater	CA	Marine Agar	Ruginescu et al., 2022	1.57	1.71	1.64	0.07	ND	ND			ND	ND		
MA CA P1.8	seawater	CA	Marine Agar	Ruginescu et al., 2022	1.79	1.78	1.78	0.01	ND	ND			ND	ND		
MA CA P3.1	seawater	CA	Marine Agar	Ruginescu et al., 2022	1.83	1.74	1.78	0.04	w+	w+			-	-		
MA CA P3.7	seawater	CA	Marine Agar	Ruginescu et al., 2022	1.31	1.38	1.34	0.03	ND	ND			ND	ND		
SWA CA P1.11	seawater	CA	eSWA	Ruginescu et al., 2022	-	-			ND	ND			ND	ND		
SWA CA P2.4	seawater	CA	eSWA	Ruginescu et al., 2022	w+	w+			ND	ND			ND	ND		
SWA CA P1.22	seawater	CA	eSWA	Ruginescu et al., 2022	w+	w+			ND	ND			ND	ND		

SWA CA P1.16	seawater	CA	eSWA	Ruginescu et al., 2022	1.70	1.79	1.74	0.04	ND	ND			ND	ND		
MA CA P1.5	seawater	CA	Marine Agar	Ruginescu et al., 2022	1.52	1.33	1.43	0.10	ND	ND			ND	ND		
SWA CA P1.4	seawater	CA	eSWA	Ruginescu et al., 2022	1.83	1.78	1.81	0.03	1.78	1.75	1.76	0.01	w+	w+		
SWA CA P1.20	seawater	CA	eSWA	Ruginescu et al., 2022	2.11	2.24	2.17	0.07	1.67	1.67	1.67	0.00	w+	w+		
SWA CA P3.5-2	seawater	CA	eSWA	Ruginescu et al., 2022	w+	w+			ND	ND			ND	ND		
SWA CA P1.10	seawater	CA	eSWA	Ruginescu et al., 2022	w+	w+			ND	ND			ND	ND		
MA EN P1.9	seawater	EN	Marine Agar	Ruginescu et al., 2022	1.50	1.56	1.53	0.03	-	-			-	-		
MA EN P3.9	seawater	EN	Marine Agar	Ruginescu et al., 2022	w+	w+			ND	ND			ND	ND		
MA EN P3.3	seawater	EN	Marine Agar	Ruginescu et al., 2022	1.54	1.67	1.60	0.06	ND	ND			ND	ND		
SWA EN P1.8	seawater	EN	eSWA	Ruginescu et al., 2022	1.22	1.24	1.23	0.01	ND	ND			ND	ND		
MA EN P2.5	seawater	EN	Marine Agar	Ruginescu et al., 2022	1.43	1.50	1.46	0.04	ND	ND			ND	ND		
MA EN P2.6 (EN45)	seawater	EN	Marine Agar	Ruginescu et al., 2022	1.44	1.44	1.44	0.00	w+	w+			2.87	2.62	2.75	0.13
SWA EN P2.3	seawater	EN	eSWA	Ruginescu et al., 2022	1.79	1.71	1.75	0.04	ND	ND			ND	ND		
MA EN P2.4	seawater	EN	Marine Agar	Ruginescu et al., 2022	1.88	1.81	1.84	0.03	-	-			-	-		
SWA EN P1.16	seawater	EN	eSWA	Ruginescu et al., 2022	w+	w+			ND	ND			ND	ND		

SWA EN P2.7	seawater	EN	eSWA	Ruginescu et al., 2022	1.50	1.50	1.50	0.00	ND	ND			ND	ND		
SWA EN P2.4	seawater	EN	eSWA	Ruginescu et al., 2022	w+	w+			ND	ND			ND	ND		
SWA EN P3.3	seawater	EN	eSWA	Ruginescu et al., 2022	-	-			ND	ND			ND	ND		
MA EN P3.8 (EN12)	seawater	EN	Marine Agar	Ruginescu et al., 2022	1.88	1.88	1.88	0.00	2.91	3.80	3.35	0.45	3.71	3.86	3.79	0.07
MA EN P2.10	seawater	EN	Marine Agar	Ruginescu et al., 2022	-	-			ND	ND			ND	ND		
SWA EN P3.5	seawater	EN	eSWA	Ruginescu et al., 2022	1.40	1.67	1.53	0.13	ND	ND			ND	ND		
SWA EN P1.14	seawater	EN	eSWA	Ruginescu et al., 2022	w+	w+			ND	ND			ND	ND		
SWA EN P3.1	seawater	EN	eSWA	Ruginescu et al., 2022	w+	w+			ND	ND			ND	ND		
SWA EN P3.6	seawater	EN	eSWA	Ruginescu et al., 2022	w+	w+			ND	ND			ND	ND		
SWA EN P2.1	seawater	EN	eSWA	Ruginescu et al., 2022	w+	w+			ND	ND			ND	ND		
SWA EN P3.4	seawater	EN	eSWA	Ruginescu et al., 2022	-	-			ND	ND			ND	ND		
MA EN P1.4	seawater	EN	Marine Agar	Ruginescu et al., 2022	1.44	1.39	1.42	0.03	ND	ND			ND	ND		

Table S6. Overall genome properties of *Stutzerimonas* sp. R75.

**Genome feature**	**Value**
Total length (bp)	4,812,634
Number of contigs	39
Largest contig (bp)	648,771
N50	228,273
N90	65,868
L50	6
L90	20
GC content (%)	60.3
Completeness (%)	100
Contamination (%)	0.14
Coding DNA sequences (CDS)	4422
Number of rRNA genes	3
Number of tRNA genes	54
Number of tmRNA genes	1

Table S7. List of enzymes homologous (>90% identity) to R75estA, R75estB, and R75estC, identified by BLASTP against the nr database (NCBI). Cells highlighted in blue represent enzymes of marine origin. Abbreviations: NS (not specified).

**Enzyme**	**GenBank Accession No.**	**Product**		**Length (aa)**	**Bacterial source**	**Origin**
**R75estA**	QWV18284.1	Alpha/beta hydrolase	Predicted dienelactone hydrolase	282	Stutzerimonas zhaodongensis A252	Ocean water, Canada: Clayoquot Sound
	RBA58146.1	Alpha/beta hydrolase	Predicted dienelactone hydrolase	282	Stutzerimonas zhaodongensis SST2	Marine sediment, Antarctica
	MFI8741923.1	Alpha/beta hydrolase		282	Pseudomonas sp. NPDC077451	NS
	ANF24624.1	Lipase	Predicted dienelactone hydrolase	282	Stutzerimonas stutzeri 273	East China Sea
	MBU0950187.1	Alpha/beta hydrolase		282	Gammaproteobacteria bacterium	Groundwater metagenome, Sweden
	KJJ64570.1	Lipase	Predicted dienelactone hydrolase	282	Pseudomonas sp. 10B238	Deep-sea sediment, China: the South China Sea
	HBM08833.1	Alpha/beta hydrolase		282	Pseudomonas sp. UBA9693	Marine Metagenome
	MBK3793917.1	Alpha/beta hydrolase		282	Stutzerimonas stutzeri KOL14.W.20.2	Submarine volcano, Greece: Santorini, Kolumbo
	MBK3875407.1	Alpha/beta hydrolase		282	Stutzerimonas stutzeri KOL14.W.20.3	Submarine volcano, Greece: Santorini, Kolumbo
	PKG94668.1	Alpha/beta hydrolase	Predicted dienelactone hydrolase	282	Pseudomonas sp. Choline-3u-10	Sea ice, USA: near Point Barrow, Alaska
	AZZ47096.1	Alpha/beta hydrolase	Predicted dienelactone hydrolase	282	Pseudomonadaceae bacterium SI-3	Host: Ulva prolifera, China: the Yellow Sea
	MCQ4281871.1	Alpha/beta hydrolase		282	Stutzerimonas stutzeri MT-1	Mariana Trench, USA: Mariana islands
	HAB64855.1	Alpha/beta hydrolase		282	Pseudomonas sp. UBA12232	Terrestrial metagenome
	MCQ4260742.1	Alpha/beta hydrolase		282	Stutzerimonas stutzeri A563/77	Clinical sample, United Kingdom
	XKY15949.1	Alpha/beta hydrolase		282	Stutzerimonas zhaodongensis JZ60	Plant: Panicum turgidum, Saudi Arabia: Jizan
	MFG3452420.1	Alpha/beta hydrolase		282	Stutzerimonas stutzeri NPDC047943	NS
	WP_181297663.1	Alpha/beta hydrolase	Predicted dienelactone hydrolase	282	Pseudomonas sp. Q2-TVG4-2	Deep-sea sediment, Atlantic Ocean: North Atlantic

	MCQ4306809.1	Alpha/beta hydrolase		282	Stutzerimonas stutzeri 28a39	Soil, Israel
	MCQ4289189.1	Alpha/beta hydrolase		282	Stutzerimonas stutzeri SD25545	Clinical sample, Palma de Mallorca
	WP_222909811.1	Alpha/beta hydrolase	Predicted dienelactone hydrolase	282	Pseudomonas sp. DNDY-54	Deep-sea sediment, Ninetyeast Ridge
	WP_404438676.1	Alpha/beta hydrolase		282	Stutzerimonas chloritidismutans AW-1	Biomass of an anaerobic chlorate-reducing bioreactor
	WP_243604255.1	Alpha/beta hydrolase	Predicted dienelactone hydrolase	282	Pseudomonas marianensis PS1	Deep-sea sediments of the Mariana Trench
	MCQ4278436.1	Alpha/beta hydrolase	Predicted dienelactone hydrolase	282	Stutzerimonas stutzeri 4C29	Marine sediment, Germany: Dangast
**R75estB**	QWV15658.1	arylesterase	Lysophospholipase_L1_like	201	Stutzerimonas zhaodongensis A252	Ocean water, Canada: Clayoquot Sound
	RBA54988.1	arylesterase	Lysophospholipase_L1_like	201	Stutzerimonas zhaodongensis SST2	Marine sediment, Antarctica
	MFI8739753.1	arylesterase		201	Pseudomonas sp. NPDC077451	NS
	MBU0948384.1	arylesterase		201	Gammaproteobacteria bacterium	Groundwater metagenome, Sweden
	KJJ62129.1	esterase	Lysophospholipase_L1_like	201	Pseudomonas sp. 10B238	Deep-sea sediment, China: the South China Sea
	MBK3795307.1	arylesterase		201	Stutzerimonas stutzeri KOL14.W.20.2	Submarine volcano, Greece: Santorini, Kolumbo
	HBM10879.1	arylesterase		201	Pseudomonas sp. UBA9693	Marine metagenome
	MBK3878338.1	arylesterase		201	Stutzerimonas stutzeri KOL14.W.20.3	Submarine volcano, Greece: Santorini, Kolumbo
	PKG95068.1	arylesterase	Lysophospholipase_L1_like	201	Pseudomonas sp. Choline-3u-10	Sea ice, USA: near Point Barrow, Alaska
	ANF28140.1	arylesterase		201	Stutzerimonas stutzeri 273	East China Sea
	AZZ47671.1	arylesterase	Lysophospholipase_L1_like	201	Pseudomonadaceae bacterium SI-3	Host: Ulva prolifera, China: the Yellow Sea
	MCQ4283062.1	arylesterase		201	Stutzerimonas stutzeri MT-1	Mariana Trench, USA: Mariana islands
	UNG17001.1	arylesterase	Lysophospholipase_L1_like	201	Stutzerimonas zhaodongensis LQ01-5	Sediment: Indian Ocean
	MAL34775.1	arylesterase		201	Pseudomonas sp. IN2	Marine water metagenome: South Pacific Ocean
	MCQ4320012.1	arylesterase		201	Stutzerimonas stutzeri CLN100	Chemical industry waste water, Germany

	MDX2352307.1	arylesterase		201	Stutzerimonas xanthomarina PP-22	Marine sponge reef biome, Ireland: North Atlantic
	VXC64444.1	multifunctional acyl-CoA thioesterase I and protease I and lysophospholipase L1		201	Pseudomonas sp. 9Ag	Host: Marine algae
	MCP9338823.1	arylesterase		201	Stutzerimonas xanthomarina DSM 18231	Host: Halocynthia aurantium, Japan: sea of Japan
	HBS76999.1	arylesterase		201	Pseudomonas sp. UBA9687	Marine metagenome
	HCC61325.1	arylesterase		201	Pseudomonas sp. UBA10494	Marine sediment metagenome
	MBK58468.1	arylesterase		201	Pseudomonas sp. SP291	Marine water sample, Indian Ocean: Northwest Arabian Sea
	MCQ4279682.1	arylesterase		201	Stutzerimonas stutzeri 4C29	Marine sediment, Germany: Dangast
	WP_342406888.1	arylesterase		201	Stutzerimonas chloritidismutans	NS
	TYP61434.1	acyl-CoA thioesterase-1	acyl-CoA thioesterase-1	201	Stutzerimonas stutzeri NP_8Ht	NS
	MCH2340144.1	arylesterase		201	Pseudomonas sp. ALOHA_A2.5_105	Ocean water sample from Hawaii Ocean
**R75estC**	QWV16884.1	Alpha/beta fold hydrolase	Predicted esterase	218	Stutzerimonas zhaodongensis A252	Ocean water, Canada: Clayoquot Sound
	RBA53873.1	Alpha/beta fold hydrolase	Predicted esterase	218	Stutzerimonas zhaodongensis SST2	Marine sediment, Antarctica
	MFI8742901.1	Alpha/beta fold hydrolase		218	Pseudomonas sp. NPDC077451	NS
	HAB62669.1	Carboxylesterase		218	Pseudomonas sp. UBA12232	Terrestrial metagenome
	AZZ44429.1	Carboxylesterase	Predicted esterase	218	Pseudomonadaceae bacterium SI-3	Host: Ulva prolifera, China: the Yellow Sea
	ANF26186.1	Carboxylesterase	Predicted esterase	218	Stutzerimonas stutzeri 273	East China Sea
	UNG19817.1	Alpha/beta fold hydrolase	Predicted esterase	218	Stutzerimonas zhaodongensis LQ01-5	Sediment: Indian Ocean
	MCQ4282401.1	Alpha/beta fold hydrolase		218	Stutzerimonas stutzeri MT-1	Mariana Trench, USA: Mariana islands
	KJJ63211.1	Carboxylesterase	Predicted esterase	218	Pseudomonas sp. 10B238	Deep-sea sediment, China: the South China Sea
	MBK3796364.1	Alpha/beta fold hydrolase		218	Stutzerimonas stutzeri KOL14.W.20.2	Submarine volcano, Greece: Santorini, Kolumbo

	MBK3876867.1	Alpha/beta fold hydrolase		218	Stutzerimonas stutzeri KOL14.W.20.3	Submarine volcano, Greece: Santorini, Kolumbo
	PKG94390.1	Carboxylesterase	Predicted esterase	218	Pseudomonas sp. Choline-3u-10	Sea ice, USA: near Point Barrow, Alaska
	MBU0949103.1	Alpha/beta fold hydrolase		218	Gammaproteobacteria bacterium	Groundwater metagenome, Sweden
	MAL37098.1	Carboxylesterase		218	Pseudomonas sp. IN2	Marine water metagenome: South Pacific Ocean
	HBM06555.1	Carboxylesterase		218	Pseudomonas sp. UBA9693	Marine metagenome
	MCQ4277660.1	Alpha/beta fold hydrolase		218	Stutzerimonas stutzeri 4C29	Marine sediment, Germany: Dangast
	WP_342405376.1	Alpha/beta fold hydrolase	Predicted esterase	218	Stutzerimonas chloritidismutans AW-1	Biomass of an anaerobic chlorate-reducing bioreactor
	HAQ88734.1	Carboxylesterase		218	Pseudomonas sp. UBA9381	Marine metagenome
	MBK3847889.1	Alpha/beta fold hydrolase		218	Stutzerimonas xanthomarina KOL14.W.430.4	Submarine volcano, Greece: Santorini, Kolumbo
	MDX2351845.1	Alpha/beta fold hydrolase		218	Stutzerimonas xanthomarina PP-22	Marine sponge reef biome, North Atlantic
	VXC89751.1	Carboxylesterase 2	Predicted esterase	218	Pseudomonas sp. 9Ag	Host: Marine algae
	MCQ4319919.1	Alpha/beta fold hydrolase		218	Stutzerimonas stutzeri	Chemical industry waste water, Germany
	SHH18422.1	Carboxylesterase	Predicted esterase	218	Stutzerimonas xanthomarina DSM 18231	Host: Halocynthia aurantium, Sea of Japan
	MCH2341107.1	Alpha/beta fold hydrolase		218	Pseudomonas sp. ALOHA_A2.5_105	Ocean water sample from Hawaii Ocean, metagenome
	MCQ4258474.1	Alpha/beta fold hydrolase		218	Stutzerimonas stutzeri A563/77	Clinical sample, United Kingdom
	MCQ2029122.1	Alpha/beta fold hydrolase		218	Stutzerimonas zhaodongensis PE	Putidoil, Spain: Mallorca
	RMH90271.1	Alpha/beta fold hydrolase	Predicted esterase	218	Stutzerimonas zhaodongensis NEAU-ST5-21	Saline-alkaline soil: Zhaodong City, China
